# TLR agonists as adjuvants for viral vaccines: mechanisms, applications, and future directions

**DOI:** 10.3389/fmicb.2025.1740572

**Published:** 2026-01-06

**Authors:** Fengyan Shao, Xiangyu Zhu, Ming Yi, Hongyu Gao, Jiali Wu, Ruikang Fang, Yubiao Xie, Jicheng Han, Huijun Lu

**Affiliations:** 1 College of Veterinary Medicine, Jilin Agricultural University, Changchun, Jilin, China; 2 Key Laboratory of Jilin Province for Traditional Chinese Medicine Prevention and Treatment of Infectious Diseases, College of Integrative Medicine, Changchun University of Chinese Medicine, Changchun, Jilin, China; 3 State Key Laboratory of Pathogen and Biosecurity, Key Laboratory of Jilin Province for Zoonosis Prevention and Control, Changchun Veterinary Research Institute, Chinese Academy of Agricultural Sciences, Changchun, Jilin, China; 4 Changchun Zhuoyi Biological Co., Ltd., Changchun, Jilin, China; 5 Jiangsu Co-lnnovation Center for the Prevention and Control of Important Animal Infectious Discases and Zoonoses, Yangzhou University, Yangzhou, Jiangsu, China

**Keywords:** adaptive immunity, immunomodulation, innate immunity, TLR agonists, toll-like receptors (TLRs), vaccine adjuvants, viral vaccines

## Abstract

Toll-like receptors (TLRs) play a pivotal role in the innate immune system by recognizing pathogen-associated molecular patterns (PAMPs) and damage-associated molecular patterns (DAMPs), thereby initiating immune responses against viral infections. TLR agonists have emerged as promising adjuvants to enhance the efficacy of viral vaccines by modulating immune responses, improving antigen presentation, and promoting both humoral and cellular immunity. This review comprehensively summarizes the classification, signaling mechanisms, and immunomodulatory functions of cell-surface and intracellular TLRs. It further discusses the application of TLR agonists as adjuvants in vaccines against major viruses, including HBV, HCV, HIV, SARS-CoV-2, influenza, and flaviviruses. Key findings from preclinical and clinical studies highlight the potential of TLR agonists to overcome immune tolerance, enhance vaccine immunogenicity, and provide broad-spectrum protection. Finally, it points toward the “integration of precision adjuvants with novel vaccine platforms” as a core future direction, laying a theoretical and applied foundation for TLR agonists to become the next generation of viral vaccine adjuvants.

## 1 Introduction

Toll-like receptors (TLRs) play a crucial role in the innate immune system, where they act as the first line of defense against invading pathogens. TLRs recognize pathogen-associated molecular patterns (PAMPs) found on pathogens to initiate immune responses to fight an infection. TLRs also recognize damage-associated molecular patterns (DAMPs) released during tissue damage, triggering responses to sterile inflammation. Many types of viruses have been shown to trigger innate immunity via TLRs. The induction of these immune responses reflects the host’s ability to rapidly recognize pathogens and mount effective defenses, while also laying the groundwork for initiating subsequent adaptive immune responses (Finberg and Kurt-Jones, 2004; Holze et al., 2024). Therefore, TLRs may be a potential adjuvant for viral vaccines.

Vaccine adjuvants are constituents added to vaccine formulations to enhance the immune response to vaccine antigens. They can increase the level and quality of antibody production, enhance cellular immune responses, thereby reducing the required antigen dose and number of vaccinations. Vaccine adjuvants are mainly categorized into mineral salts (e.g., alum), oil-in-water emulsions (e.g., MF59, containing squalene, Tween 80, and Span 85), liposomes, proteins (e.g., cytokines), nucleic acids (e.g., CpG oligodeoxynucleotides), and combination adjuvants. TLR agonists, as nucleic acid-based adjuvants, possess the properties of traditional adjuvants but their core advantage lies in their ability to precisely shape the type of immune response by targeted modulation of signaling pathways (Ravikumar et al., 2022). However, the quantitative relationship between TLR signal strength and the formation of immune memory remains controversial within the field: some studies suggest that sustained weak activation favors memory B cell generation (Awate et al., 2013), while others confirm that short-term strong stimulation can induce long-lasting CTL memory. This contradiction highlights the complexity of dose- and time-dependent effect modulation in TLR adjuvant applications (Hawkins et al., 2013; Smyth et al., 2013).

To date, ten functional TLRs in human (TLR1-10) and twelve in mice (TLR1-9, 11–13) have been identified (Liao et al., 2017). TLRs can be classified into two categories based on the location of their expression: cell surface TLRs and intracellular TLRs. The former primarily recognize lipid and protein ligands, while the latter focuses on nucleic acid ligands. TLRs agonists include natural and synthetic TLRs and different TLRs have been identified with specific natural ligands. These ligands include lipoproteins and peptidoglycans for TLR2, double-stranded RNA (viral) for TLR3, bacterial lipopolysaccharides (LPS) and lipophosphatidic wall acids for TLR4, bacterial flagellin for TLR5, single-stranded RNA for TLR7 and TLR8, unmethylated CpG motifs found in bacterial DNA or viruses for TLR9, and viral proteins/viral RNP complexes for TLR10 (Weeratna et al., 2005; Fore et al., 2020; Owen et al., 2020). Thus, TLRs have great potential as viral vaccine adjuvants.

There are five main subfamilies of TLRs, which are classified into TLR2 family, TLR3 family, TLR4 family, TLR5 family, and TLR9 family based on differences in their amino acid sequences and genome structures; the TLR2 family includes TLR1, TLR2, TLR6, and TLR10; the TLR4 family and the TLR5 family comprise of single receptors, i.e., TLR4 and TLR5, respectively; while the TLR9 family consists of TLR7, TLR8 and TLR9 (Kawasaki and Kawai, 2014). TLRs can be found on the cell membrane of immune cells such as macrophages, dendritic cells, T cells and B cells. These cells play important roles in the immune system, and the presence of TLRs provides them with the ability to rapidly recognize and respond to pathogen invasion (Chen et al., 2014).

## 2 Association between TLR signaling pathways and downstream immune functions

**Figure 1:**
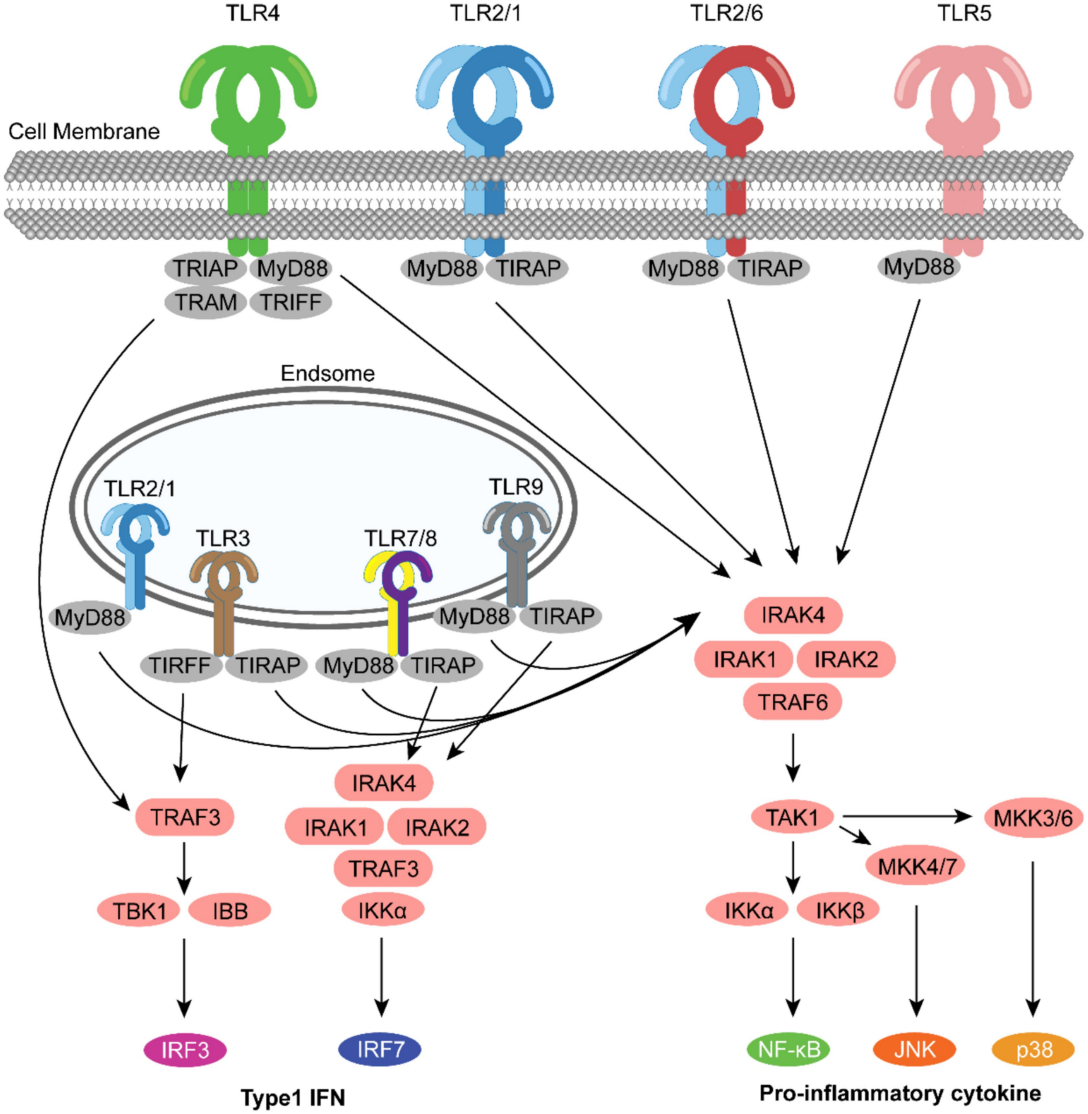
Localization of TLRs and various TLR signaling pathways. TLRs were divided into two categories: cell surface TLRs and intracellular TLRs. TLR1, TLR2, TLR4 and TLR6 interact with hydrophobic ligands (i.e., lipopeptides, lipopolysaccharides), while TLR3, TLR7, TLR8 and TLR9 are activated by hydrophilic molecules (i.e., proteins and nucleic acids). TLRs are trans membrane proteins that consist of a toll/interleukin-1 receptor (TIR) domain that upon ligand-induced dimerization, can transmit intracellular signals via the recruitment adaptor proteins. MyD88 is a universal adaptor protein for all TLRs with the exception of TLR3, and through activation of the interleukin-1 receptor-associated kinases (IRAKs)/TNF receptor-associated factor (TRAF) 6 complex, TLRs can activate AP1 and NF-κB pathways leading to the expression of proinflammatory cytokines. TLR3 (and TLR4), through TIR-domain-containing adapter-inducing interferon (TRIF), and TLR7 and TLR9, through MyD88, can activate interferon-regulatory factors (IRFs) 3 and 7 via TRAF3 leading to a type 1 interferon response. Other TLR adaptors include MyD88 adaptor-like (MAL), also known as TIR domain-containing adaptor protein (TIRAP), used for TLR2 and TLR4 MyD88-dependent signaling and TRIF-related adaptor molecule (TRAM) for TRIF signaling.

TLRs can be classified into two categories based on the location of their expression: cell surface TLRs and intracellular TLRs. These TLRs include different isoforms that can recognize ligands to activate the corresponding signaling pathways to perform immunomodulatory functions (Owen et al., 2020). TLR agonists activate downstream signaling pathways by binding to TLRs, which triggers a series of immune responses. These include the recruitment of molecules such as myeloid differentiation factor 88 (MyD88) and TIR structural domain-containing adaptor protein (TIRAP)/MyD88 articulin-like protein (MAL). The Toll-Interleukin receptor (TIR) domain-containing adaptor protein inducing IFNβ (TRIF, also known as TICAM1) and TRIF-related adaptor molecule (TRAM). TIR and TRAM are known to activate the transcription factors NF-κB or Interferon Regulatory Factors (IRF) (Alexopoulou and Irla, 2025) (Figure 1).

### 2.1 Immunoregulatory division of core signaling pathways

The MyD88-dependent pathway is ubiquitous in all TLRs except TLR3. Upon activation, it triggers the IRAK-TRAF6-TAK1 cascade, activating the NF-κB and MAPK pathways. NF-κB primarily regulates the expression of pro-inflammatory cytokines like IL-6 and TNF-*α*, while the MAPK pathway (p38, JNK, ERK) influences cell proliferation and differentiation by regulating the AP-1 transcription factor (Zhu et al., 2025; Shang et al., 2025). Recent studies confirm that the phosphorylation level of IRAK4 in the MyD88 pathway is a key node regulating Th1/Th2 polarization: low-level phosphorylation promotes IL-4 production (Th2 bias) (Feng et al., 2025), while high-level phosphorylation induces IFN-*γ* secretion (Th1 bias) (Zhang et al., 2025), This discovery provides a molecular target for the immune-directional design of TLR adjuvants.

The TRIF-dependent pathway primarily exists in TLR3 and TLR4, inducing type I interferon (IFN-*α*/*β*) production via the TBK1-IRF3 pathway (Hu et al., 2024). Unlike the pro-inflammatory effects of the MyD88 pathway, the TRIF pathway focuses more on activating the antiviral functions of dendritic cells (DCs) and NK cells (Kumar, 2020). TLR4 can activate both pathways simultaneously, with the MyD88 pathway initiating within 15 min post-activation and the TRIF pathway delayed until about 30 min later. This temporal difference may explain why TLR4 adjuvants exhibit both pro-inflammatory and antiviral effects (Richard et al., 2020; Kuriakose et al., 2014). Notably, excessive activation of TLR4 signaling during acute viral infection can also lead to harmful hyperinflammation (He et al., 2025).

### 2.2 Regulatory effects on key immune cell functions

#### 2.2.1 Th1 and Th2 polarization regulation

Different TLR agonists directionally regulate T helper cell differentiation through selective activation of signaling pathways: TLR3, a potent driver of Th1 responses, activates the TBK1-IRF3 axis via its unique TRIF-dependent pathway, inducing robust production of type I interferons (IFN-*α*/*β*). This signaling cascade concurrently activates NF-κB, which synergistically promotes interleukin-12 (IL-12) secretion by dendritic cells (DCs) (Jack et al., 2007; Hsieh et al., 2025). TLR7/8/9 agonists primarily induce DCs to secrete IL-12 via the MyD88-IRF7 pathway, thereby activating Th1 cells; whereas TLR2/5 agonists promote IL-4 production through the MyD88-NF-κB pathway, favoring Th2 polarization (Sugitharini et al., 2016; Didierlaurent et al., 2004).

#### 2.2.2 Regulation of Th17 and Tfh cells

Selected TLR agonists (e.g., TLR2/4/5 agonists), under specific cytokine milieus (such as the presence of TGF-*β* and IL-6), can induce the differentiation of naïve T cells into Th17 cells (Uematsu and Akira, 2009). This process is dependent on the master transcription factor RORγt and leads to the production of effector cytokines IL-17A/F and IL-22, which play a pivotal role in combating extracellular bacterial and fungal infections, as well as in mediating autoimmune pathogenesis (Manni et al., 2011). In contrast, TLR7/8/9 agonists effectively promote the production of IL-6 and IL-21, thereby inducing the expression of the transcription factor Bcl-6 and driving Tfh cell differentiation (Ribeiro et al., 2023; Ugolini et al., 2018). Tfh cells are characterized by their follicular homing capacity. Through providing co-stimulatory signals and secreting IL-21, they professionally help B cells to accomplish antibody class switching, affinity maturation, and the formation of germinal centers, making them essential for high-efficacy humoral immunity (Qi et al., 2014; Yang et al., 2023).

#### 2.2.3 Cytotoxic T lymphocyte (CTL)

The cytotoxic activity of CTLs is crucial for clearing virus-infected cells. TLR agonists primarily regulate CTL function through two pathways: “enhanced DC cross-presentation” and “synergistic NK cell activation.” TLR7/8 agonists can induce high expression of CD8α molecules on DCs, improving their cross-presentation efficiency (Sanlorenzo et al., 2025); simultaneously, the TLR5 agonist CBLB502 activates NK cells to secrete IFN-*γ*, enhancing CTL responses (Wang et al., 2023).

#### 2.2.4 Regulatory T cells (TREG)

Regulatory T cells play a pivotal role in maintaining immune tolerance and preventing autoimmunity and excessive inflammation. However, in vaccine immunology, an overly potent TREG response can potentially suppress the desired protective immunity. TLR agonists exert a complex and bidirectional regulatory influence on TREG, an effect determined by the specific TLR involved, dosage, and the immunological microenvironment (Guo et al., 2020; Kubota et al., 2010).

On one hand, certain TLR agonists can inhibit TREG function or differentiation. For instance, the TLR8 agonist has been demonstrated to directly recognize and dismantle the Foxp3 protein within human TREG cells, thereby specifically abrogating their immunosuppressive function. This mechanism provides a novel strategy for breaking immune tolerance in cancer immunotherapy. On the other hand, some TLR agonists may induce or enhance TREG responses under specific conditions. Although typically associated with Th2 responses, the TLR2 agonist can, in certain microenvironments, enhance TREG proliferation and immunosuppressive function, highlighting the complexity of its actions (Sutmuller et al., 2006). Meanwhile, the TLR4 agonist MPLA, acting as a low-toxicity adjuvant, not only induces a robust Th1 response but also moderately promotes the generation of IL-10-producing type 1 regulatory T (Tr1) cells. This activity helps establish a fine-tuned equilibrium between effective immunity and inflammatory control, thereby mitigating immunopathological damage (Komai-Koma et al., 2021; Tuomela et al., 2025; Yeh et al., 2013).

#### 2.2.5 B cell activation and antibody regulation

TLR agonists regulate B cell function through both direct and indirect mechanisms: the direct mechanism involves binding of agonists to TLRs on the B cell surface (e.g., TLR4, TLR7/9), activating the NF-κB pathway and promoting proliferation (Carsetti, 2025); the indirect mechanism involves BAFF and IL-21 secreted by DCs enhancing B cell affinity maturation (Robinson et al., 2019). The TLR9 agonist CpG ODN significantly promotes IgG2a class switching in B cells (Lin et al., 2004), while the TLR4 agonist MPLA preferentially induces IgG1 production. This difference in antibody subclass is closely related to the vaccine’s protective profile—IgG2a is more advantageous against viral infections, whereas IgG1 plays a prominent role in humoral immunity (Dubuske et al., 2019).

#### 2.2.6 Dendritic cell maturation and antigen presentation

As the central cells linking innate and adaptive immunity, the maturation state of DCs directly determines vaccine immunogenicity. TLR agonists enhance DC function through the following mechanisms: (a) Upregulating the expression of MHC-I/II and co-stimulatory molecules (CD80/CD86), improving antigen presentation efficiency (Zhou et al., 2025); (b)Promoting DC migration to draining lymph nodes, increasing opportunities for T cell contact (Schmidt et al., 2026); (c) Modulating the cytokine secretion profile of DCs, directionally guiding T cell differentiation (Pudjohartono et al., 2025).

#### 2.2.7 Immunoregulatory mechanisms of TLRs in macrophages

Within the central innate immune system, TLR agonists serve as master regulators that determine macrophage polarization fate. Agonists of TLR3, 4, 7, and 9 potently drive macrophages toward a classically activated M1 phenotype by activating the NF-κB and IRF signaling pathways. This polarization leads to the high expression of pro-inflammatory cytokines (IL-6, TNF-*α*, IL-12) and confers potent antimicrobial and antitumor capabilities (Ogawa et al., 2021; Murray et al., 2014). In contrast, TLR2 agonists preferentially promote the production of the anti-inflammatory cytokine IL-10 via the MyD88-dependent pathway, thereby inducing macrophage differentiation into an alternatively activated M2 phenotype. This M2 state is subsequently involved in immunosuppression, tissue repair, and fibrotic processes (Xuan et al., 2020; Moreira et al., 2008). The strategy of selectively employing distinct TLR agonists to reprogram macrophage function has thus emerged as a pivotal approach in combating infections and cancer.

#### 2.2.8 Immune regulation of natural killer cells by TLRs

**Figure 2:**
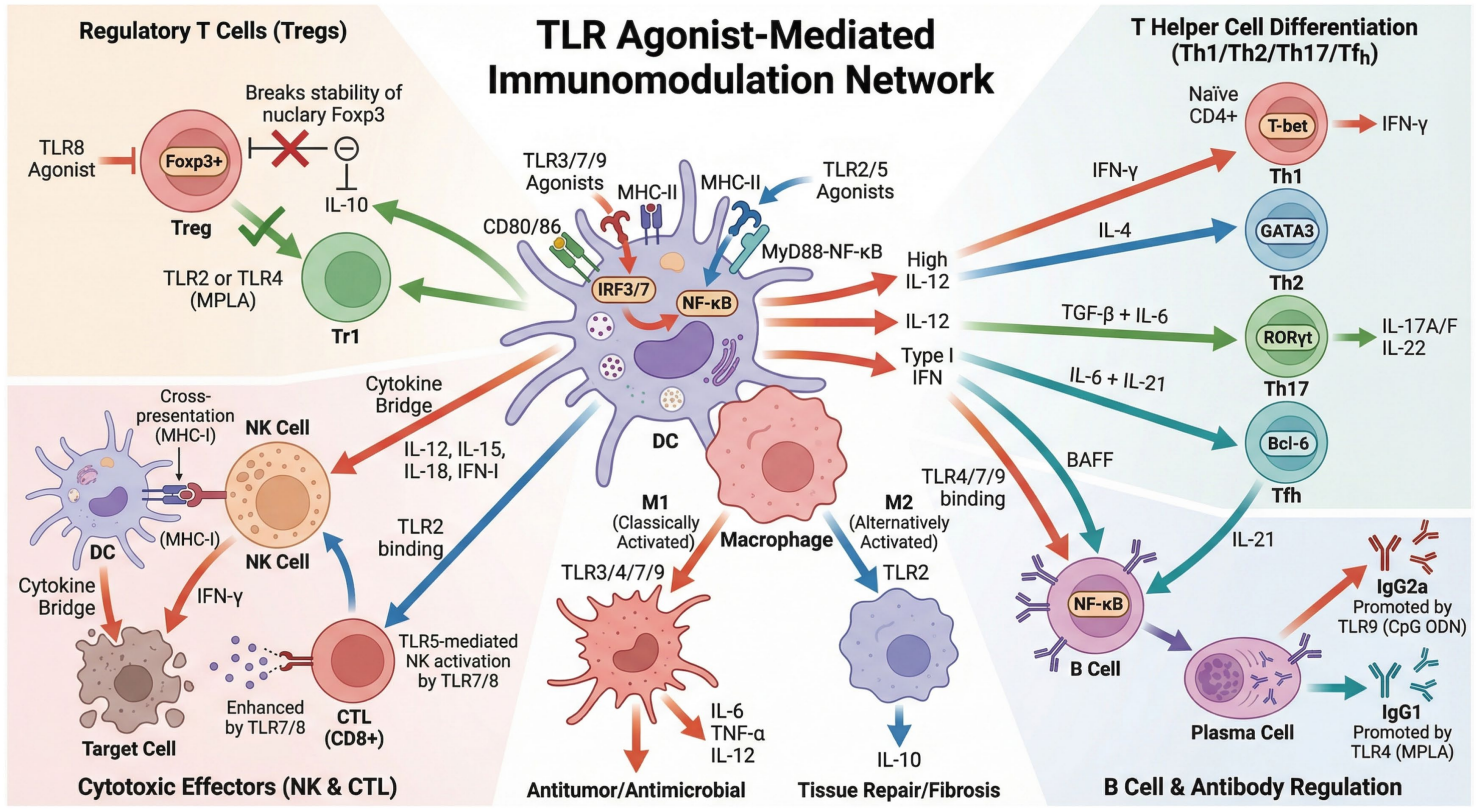
TLR agonist-mediated immunomodulation network. This illustration depicts a multidimensional regulatory network of Toll-like receptor (TLR) agonists on immune cells. Dendritic cells (DCs) serve as the central hub. Upon activation by TLR3/7/9 or TLR2/5 agonists, they secrete cytokines such as IL-12 and type I interferons via the IRF3/7 or MyD88-NF-κB signaling pathways. Concurrently, they upregulate the expression of co-stimulatory molecules and MHC-II molecules, thereby bridging innate and adaptive immunity. At the innate immunity level, TLR3/4/7/9 agonists drive macrophage polarization toward the pro-inflammatory M1 phenotype, while TLR2 agonists promote their shift toward the anti-inflammatory M2 phenotype. Natural killer (NK) cells enhance their cytotoxicity both through cytokines secreted by activated DCs and via direct stimulation by TLR7/8/5 agonists. Within the adaptive immunity arm, cytokines from DCs direct the differentiation of naïve CD4^+^ T cells into Th1, Th2, Th17, and T follicular helper (Tfh) subsets. TLR agonists concurrently regulate the stability of regulatory T cells (Tregs) and the IL-10 secretion by type 1 regulatory T (Tr1) cells. Furthermore, through direct activation and DC-mediated assistance, they promote B cell differentiation into plasma cells and facilitate antibody class-switching. Ultimately, this intricate network orchestrates a comprehensive immune response encompassing anti-tumor/anti-microbial defense, humoral immunity, and the regulation of immune homeostasis.

Toll-like receptor (TLR) agonists exert immunomodulatory effects on natural killer (NK) cells through a multi-layered and network-based precision mode. The core mechanism lies in the fact that TLR agonists are first recognized as “danger signals” by antigen-presenting cells such as dendritic cells and macrophages. This recognition induces the secretion of a core cytokine combination, primarily comprising IL-12, IL-15, IL-18, and type I interferons, which forms an efficient “cytokine bridge” that indirectly activates NK cells. This activation promotes a burst of IFN-*γ* secretion and enhances cytotoxic functions (Mahr et al., 2024; Chen et al., 2024); Concurrently, highly purified human and murine NK cells can directly recognize bacterial proteins such as FimH through the TLR4-MyD88 signaling pathway, independent of APCs. This direct engagement leads to the production of IFN-γ and TNF-*α* and augments cytotoxicity (Mahr et al., 2024; Mian et al., 2010). Based on this mechanism, novel TLR7/8 agonist nanovaccines can significantly increase intratumoral NK cell infiltration by activating APCs. Furthermore, the combination of TLR agonists with therapeutic antibodies can markedly enhance NK cell-mediated antibody-dependent cellular cytotoxicity (ADCC), providing a critical strategic foundation for anti-tumor and anti-viral immunotherapy (Veneziani et al., 2023; Dietsch et al., 2016) (Figure 2).

## 3 Potential roles of cell surface TLRs and their agonists

Cell surface TLRs initiate innate immune responses by recognizing PAMPs such as lipids, lipoproteins and proteins. Members of cell surface TLRs are TLR1, TLR2, TLR4, TLR5, TLR6 and TLR11, which recognize different pathogen components (e.g., lipoproteins, peptidoglycans, lipopolysaccharides, and flagellin) (Rolfo et al., 2023). These receptors regulate innate and adaptive immune responses by activating downstream signals via the MyD88-dependent pathway and TRIF-dependent pathway to promote the production of pro-inflammatory cytokines and type I interferons. Cell surface TLRs play important roles in infection, inflammation and autoimmune diseases and are potential targets for immunotherapy (Yamamoto and Takeda, 2010).

### 3.1 TLR1, TLR2, and TLR6 agonists: balanced immune modulators

TLR2 recognizes a diverse array of PAMPs and can form heterodimers with TLR1 and TLR6 to extend its ligand recognition specificity. The TLR1/2 or TLR2/6 heterodimer recognition ligands activate the nuclear transcription factors NF-κB and AP-1 to induce the secretion of pro-inflammatory cytokines through the MyD88 pathway (Yu et al., 2010). In recent years, research on TLR2 has intensified and includes discovery of small synthetic TLR2 agonists, modification of traditional TLR2 agonists to improve their properties and the biocoupling of TLR2 with vaccine antigens (Santone et al., 2015).

Soluble factors released by *Neisseria meningitidis* serogroup B / serotype 15 (strain H44/76) induced the IL-8 promoter in cells transfected with TLR1 and TLR2. Wyllie et al. proposed that the microbial response pattern in cells co-transfected with TLR1 and TLR2 was different from that in cells transfected with either molecule alone and later confirmed that TLR1 and TLR2 act together in association with each other (Wyllie et al., 2000). Lipids produced by *Mycoplasma mycoides* are recognized only in the presence of both TLR2 and TLR6 while the *Bacillus anthracis* toxin PGA is a newly discovered TLR2/6 agonist (Lee et al., 2015). TLR2 can also mediate host innate immunity in isolation and act as a vaccine adjuvant to activate multiple signaling pathways for immune responses.

Cen et al. synthesized a small molecule agonist of TLR1/2, SMU-Z1, which up-regulates the expression of mouse NK and CD8^+^ T cells, and has anti-tumor as well as immune adjuvant properties. SMU-Z1 has improved *in vivo* stability and molecular weight when compared to Pam3CSK4, and its activation of B cells points to its potential as an immune adjuvant. SMU-Z1 could function as a new type of Latency-Reversing Agent (LTA) to clear HIV1 viral reservoirs (Cen et al., 2019). However, Pam series agonists still dominate among the positive effects of TLR1/2/6 agonists. XS15 (Pam3CS-GDPKHPKSF) is a novel TLR1/2 agonist based on Pam3CS, created by replacing the tetra-lysine (K4) sequence of Pam3CSK4 with diuretic peptide (GDPKHPKSF), altering the conjugate’s water solubility to facilitate uptake and purification (Rammensee et al., 2019). Qiao et al. reported that an RBD-based nanoparticle vaccine, in complex with the TLR2/6 agonist Pam2CSK4, induced a significant Th1-biased immune response and enhanced the differentiation of memory T cells and follicular helper T cells (Qiao et al., 2022). Pam2CSK4 positively regulated many genes involved in leukocyte activation and proliferation, and maintained high levels of protective IgM and IgG antibodies (Lu et al., 2020). Data suggest that Pam2CSK2, which targets TLR2, is effective in tuberculosis vaccines and is the optimal adjuvant for the SARS-CoV-2 nanoparticle vaccine (Qiao et al., 2022). However, it should be noted that TLR2 agonists might induce immune non-responsiveness in immunocompromised populations. A study on CVID patients similarly showed impaired multifunctional responses (e.g., secretion of multiple cytokines) in CD8^+^ T cells after stimulation with a TLR2 agonist, possibly due to incomplete innate immune signals affecting T cell activation and effector function (Sharifi et al., 2018). This illustrates the future need to optimize the stability of TLR2 agonists to improve their efficacy.

AXA-042 is a novel synthetic polyethylene glycosylated TLR2/6 agonist that is being developed for the treatment of advanced solid tumors. AXA-042 acts through multiple cellular mechanisms including activation of pro-inflammatory innate immune responses, reduction of immunosuppressive macrophages at the tumor site, activation of dendritic cells, and the release of chemokines to promote T cell recruitment (Tran et al., 2023). TLR2 recognizes a wide range of bacterial components and a combination of TLR2/TLR9 agonists has demonstrated a significant protective effect against both Antibody (Ab) lethal and sublethal infections, and also promotes killing or phagocytosis of Ab by lung epithelial cells and macrophages cells (Scharf et al., 2010). It reported that TLR2 also responds to viral components such as human cytomegalovirus mRNA, providing a new target for the treatment of viral diseases (Lee et al., 2022). In recent years Satitsuksanoa et al. found that airborne mites contain the fatty acid-binding protein Derp13 that may play a role in the initiation of house dust mite allergic responses through its lipid-binding ability via TLR2 activation (Satitsuksanoa et al., 2016).

### 3.2 TLR4 agonists: benchmark molecules in clinical translation

TLR4 is the most studied member of the TLR family and recognizes bacterial lipopolysaccharide (LPS). TLR4 is located at the plasma membrane and is expressed predominantly on myeloid cells, but not expressed by plasmacytoid dendritic cells (pDCs) and naïve B cells. TLR4 recognizes LPS through its co-receptors myeloid differentiation factor-2 (MD-2) and CD14 (Kaur et al., 2022). Activation of the TLR4 transmembrane receptor structure involves the TIRAP-MyD88-dependent signaling pathway, which regulates NF-κB activation and the coordinated production of inflammatory cytokines, ultimately leading to the formation and functional enhancement of the IKK/NF-κB complex (Litak et al., 2020).

Some TLR4 agonists have shown promising experimental outcomes and are now in clinical trials. Morin et al. identified a new class of small molecule mouse TLR4 agonists, Neoseptins. The compounds, screened from an *α*-helical mimetic library, stimulate immune responses, have a well-defined mechanism of action, are easy to prepare and structurally modify, are non-toxic, and trigger better and qualitatively different responses than lipopolysaccharide (LPS), although they act on the same receptor (Morin et al., 2016). In terms of anti-infection, clinical trials of TLR4 agonists for certain refractory infections are underway to confirm their efficacy and safety for clinical treatment. Antonio et al. demonstrated that the TLR4 agonist Monophosphoryl Lipid A (MPLA) induced the expansion and recruitment of intrinsic leukocytes to the site of infection and was also involved in inducing metabolic reprogramming of leukocytes, which consistently enhanced macrophage glycolysis, mitochondrial function and tricarboxylic acid cycle flux. Taken together, these features support the use of TLR4 agonists as stand-alone therapeutic agents or antimicrobial adjuvants in infection-prone populations (Hernandez et al., 2019). In addition, a study combining the adjuvant MPLA and the NKT cell agonist KRN7000 demonstrated that, when applied simultaneously, it had a synergistic effect in specific immunity against the mucin-type O-glycans (Tn antigens), which can overcome the shortcomings of weak immunity against glyco-antigens and susceptibility to immune tolerance (Noh et al., 2020). In the field of oncology therapy, synthetic TLR4 agonists immune adjuvants in combination with chemotherapeutic agents and immune checkpoint inhibitors have shown good safety and efficacy (Chettab et al., 2023). It demonstrated that MPLPS (chemically detoxified LPS) formulated in liposomes has strong therapeutic potential as a systemic anticancer agent with activity and warrants further evaluation in cancer patients. Another potential therapeutic option for cancer patients is the detoxifying TLR4 agonist F1Lipo-LPS which presented with potent antitumor activity in immunocompetent models and strong adjuvant effect in combination with therapeutic monoclonal antibodies in immunodeficient models (Chettab et al., 2023). Combining TLR4 agonists and antigens with magnetic nanoparticles mimicking pathogens to enhance their uptake and processing by antigen-presenting cells, activated T cell-mediated anti-tumor immune responses. Meanwhile, TLR4 agonists in combination with programmed death ligand 1 (PD-L1) blockers significantly improved the anti-tumor effect, providing a new strategy and experimental basis for combined tumor immunotherapy (Traini et al., 2019).

### 3.3 TLR5: advantageous choice for mucosal immunity

TLR5 is an important member of the TLR family and plays a key role in the immune system. It mainly recognizes bacterial flagellin, and the TLR5-flagellin interaction rapidly initiates the immune response to defend against pathogen invasion (Ruan et al., 2022). TLR5 agonists bind to TLR5, forming TLR5 homodimers, which then recruit the MyD88 adaptor protein. This activates the NF-κB and MAPK signaling pathways, initiating immune responses (Holzapfel et al., 2022; Mohammadi Esfanjani et al., 2023).

Lee et al. confirmed that the novel TLR5 agonist KMRC011 enhances the efficacy of immune checkpoint inhibitors by activating innate immunity. In a mouse tumor model, KMRC011 combined with anti-PD-1 treatment significantly inhibited tumor growth (Lee et al., 2024). In a bacterial meningitis model, activation of TLR5 significantly reduced mortality and decreased the number of bacteria in the brain, suggesting that TLR5-activated meningeal macrophages play an important role in neonatal resistance to bacterial meningitis infection. A single dose of CBLB502 (TLR5 agonist) provided mice with optimal protection against mCMV (murine Cytomegalovirus) with significantly reduced hepatic viral load, increased number of Ly49H^+^ and Ly49D^+^ activated cytotoxic NK cells, and increased number of NK cells producing IFN-*γ*, granzyme B, and CD107a. CBLB502-induced anti-mCMV immunity was TLR-dependent, suggesting that CBLB502 indirectly activates NK cells and enhances anti-CMV immunity (Hossain et al., 2014). Meanwhile, for inflammatory bowel disease treatment, relevant clinical trials are evaluating the role of TLR5 agonists in disease remission and maintenance therapy. Xu et al. reported that KMRC011 reduced small intestinal mucosal injury in mice by down-regulating the NF-kB and NLRP3 pathways, thereby contributing to recovery (Xu et al., 2016).

### 3.4 TLR11: potential target for anti-parasitic vaccine adjuvants

TLR11 structure contains leucine-rich repeat (LRR) sequences and TIR structural domains. It recognizes specific PAMPs, such as the flagellin of Toxoplasma gondii, which in turn activates the innate immune response. Through a series of signaling pathways, the interaction between TLR11 and the agonist activates complex intracellular signaling pathways. Shokri et al. investigated the use of the TLR11 agonist as an adjuvant for immunization with *T. gondii* total lysis antigen (TLA) in infected BALB/c mice. The combination of TLR11 agonist and TLA significantly improved the efficacy of TLA and shifted the immune system toward the Th1 type by increasing the production of IFN-*γ* and IL-5. This simultaneously increased the proliferation of lymphocytes and the survival of *T. gondii*-infected mice, which provided strong justification for the use of the TLR11 agonist as an adjuvant in T. gondii vaccines (Shokri et al., 2020). The TLR11 signaling pathway is mostly mediated through the MyD88-dependent signaling pathway. MyD88 recruits downstream kinases such as IRAK1, IRAK4, etc., which sequentially phosphorylate and activate TRAF6, which then triggers a series of cascading reactions leading to the activation of transcription factors such as NF-κB and MAPK. This, in turn, prompts immune cells to initiate gene transcription and protein synthesis of co-stimulatory molecules such as CD80, CD86, etc. These molecules promote the proliferation and differentiation of T cells, including the different subpopulations of Th1, Th2, Th17, etc., and regulate the type and intensity of immune responses (Hatai et al., 2016; Aylsworth et al., 2013). Table 1 outlines the potential role of cell surface agonists.

**Table 1:** Potential role of cell surface agonist.

Agonist	Species	Role	References
SMU-Z1	TLR1/2 agonist	Up-regulates NK and CD8^+^ T cell expression, has anti-tumor and immune-adjuvant properties, better molecular stability, and is expected to be a novel LRA for HIV-1 viral reservoir clearance.	Cen et al. (2019)
XS15	TLR1/2 agonist	Pam3CS lipopeptide-based agonist with enhanced water solubility and uptake efficiency to promote immune cell activation.	Rammensee et al. (2019)
Pam2CSK4	TLR2/6 agonist	Induces significant Th1-biased immune responses, enhances memory T cell and follicular helper T cell differentiation, and maintains high levels of protective antibodies IgM and IgG.	Lu et al. (2020)
AXA-042	TLR2/6 agonist	Activates pro-inflammatory immune responses, reduces immunosuppressive macrophages at the tumor site, activates dendritic cells, and promotes T-cell recruitment for the treatment of advanced solid tumors.	Tran et al. (2023)
Neoseptins	TLR4 agonist	Stimulate immune response, clear mechanism of action, non-toxic, triggers different response with LPS, used in anti-infection and tumor therapy.	Morin et al. (2016)
MPLA	TLR4 agonist	Promotes the expansion and recruitment of intrinsic leukocytes to the site of infection, enhances macrophage metabolism, used as immune adjuvant in combination with chemotherapeutic agents.	Hernandez et al. (2019)
KMRC011	TLR5 agonist	Activates innate immunity, enhances the efficacy of immune checkpoint inhibitors, inhibits tumor growth and is used in the treatment of inflammatory bowel disease.	Lee et al. (2024)
CBLB502	TLR5 agonist	Enhances anti-CMV immunity, increases NK cell number, enhances IFN-*γ* and granzyme B expression, used in anti-infection therapy.	Hossain et al. (2014) and Xu et al. (2016)

## 4 Potential roles of intracellular TLRs and their agonists

Intracellular TLRs are mainly localized in membrane structures such as endosomes and lysosomes within the cell, and mainly include TLR3, TLR7, TLR8, TLR9 and TLR13. Intracellular TLRs are responsible for the recognition of nucleic acids of pathogen origin such as double-stranded RNA (TLR3), single-stranded RNA (TLR7 and TLR8), and single-stranded DNA containing unmethylated CpG motifs (TLR9) (Blasius and Beutler, 2010). These receptors activate downstream signaling pathways through their intracellular TIR structural domains, including MyD88-dependent and TRIF-dependent pathways, which promote the production of type I interferons and pro-inflammatory cytokines that exert antiviral and anti-bacterial immune effects. Intracellular TLRs play a key role in innate immune responses and are also involved in the regulation of adaptive immune responses. In addition, their aberrant activation in autoimmune diseases may be contributing to autoimmune reactions (Sameer and Nissar, 2021).

### 4.1 TLR3 agonists: “broad-spectrum weapons” against viruses

TLR3 is a transmembrane protein that is mainly expressed on the surface of immune cells, such as macrophages and dendritic cells. The key function of TLR3 is to recognize double-stranded RNA (dsRNA). When a virus infects a cell and replicates, the dsRNA produced is recognized by TLR3, which triggers an antiviral immune response, and it is an important bridge that connects innate and adaptive immunity (Fitzgerald and Kagan, 2020). TLR3 agonists activate the TRIF-dependent signaling pathway by recognizing dsRNA. They initiate the NF-κB and MAPK pathways, contributing to the production of pro-inflammatory cytokines. In immunomodulation, TLR3 agonists can enhance antiviral immune responses, activate immune cells such as dendritic cells and promote their maturation and antigen-presenting functions, thereby enhancing adaptive immune responses and playing a role in immune memory formation (Matsumoto et al., 2011). In antitumor therapy, TLR3 agonists such as Poly(I:C) and its derivatives inhibit tumor growth by activating the immune system, promote effector T-cell infiltration, and enhance the efficacy of immune checkpoint blockade therapy. However, the role of TLR3 in different cancers is two-pronged and over-activation may lead to inflammatory responses and tissue damage (Salaun et al., 2006).

TLR3 agonists have been extensively studied for use in a variety of viral vaccines, including influenza virus, HIV, Zika virus, and SARS-CoV-2. Poly(I:C), a synthetic double-stranded RNA, is the most commonly studied TLR3 agonist. It activates TLR3 and induces the production of type I interferon and other cytokines. Poly(I:C) has been widely used in vaccine research, including influenza vaccines, HIV vaccines, and tumor vaccines (Lee et al., 2022). Thomas et al. studied the adjuvant effect of poly(I:C) in a porcine influenza vaccine, confirming that when combined with the influenza vaccine, it significantly enhanced antigen-specific CD8^+^ T cell responses and improved vaccine immunogenicity (Thomas et al., 2015). With regards to the anti-tumor potential of intracellular TLRs, Poly(I:C) has been reported to stimulate the anti-tumor effect of NK cells by activating the TLR3 signaling pathway (Stowell et al., 2009). In addition to Poly(I:C), other TLR3 agonists are under investigation, such as natural dsRNA viral fragments or synthetic analogs. These agonists enhance the immune effect of vaccines by activating the TLR3 signaling pathway. Researchers have developed a novel dsRNA-based TLR3 agonist called NexAvant (NVT) It is a 275-kDa homogeneous molecule that is highly stable, and its appearance, concentration and molecular size were unaffected after 6 months under accelerated storage conditions. NVT will be a promising adjuvant for antiviral or anticancer vaccines (Ko et al., 2023). In addition, TLR3 agonists can be used in combination with other adjuvants or therapeutic approaches. Lu et al. explored the potential of aluminum hydroxide adjuvant (AH) to adsorb Poly(I:C), and the TLR9 agonist, CpG, and compared the effects of the combination of adjuvants with TLR agonists or AH alone on the immune response in mice. The results demonstrated that the formulation of aluminum hydroxide adjuvant with the TLR3 agonist Poly(I:C) and CpG enhanced the strength and affinity of the humoral immune response (Lu et al., 2019). This co-adjuvant is a promising formulation to address some of the unmet needs of current vaccines.

### 4.2 TLR7/8 agonists: core adjuvants for viral and cancer vaccines

TLR7 is mainly expressed by immune cells such as pDCs, monocytes, and B cells. It recognizes endogenous or exogenous ligands such as viral single-stranded RNA. Upon ligand binding, it activates NF-κB/MAPK signaling and can also induce type I interferons, eliciting antiviral immunity (Luo et al., 2019; Hemmi et al., 2002; Abaidullah et al., 2021). TLR8 is highly homologous to TLR7, recognizes viral ssRNA, bacterial mRNA, etc., and upon activation, initiates downstream cascades via the MyD88 pathway, promoting NF-κB-mediated inflammatory cytokine expression. It also regulates cellular stress and activates IRF7 to induce type I interferons. Both rely on the MyD88 pathway and are core targets for activating antiviral immunity and developing RNA virus vaccine adjuvants (Martínez-Espinoza and Guerrero-Plata, 2022; Vidya et al., 2018).

Imiquimod is a synthetic small molecule compound and one of the first TLR7 agonists discovered. It is commonly used clinically for the treatment of external genital and perianal condyloma acuminatum, a superficial basal cell carcinoma. Imiquimod can induce the production of a variety of cytokines, activate immune cells, enhance immune defense, and function as an antiviral and antitumor agent (Skinner, 2003). The oral TLR7 agonist GS-9620, in a Phase II clinical trial (NCT03650235) for chronic HBV patients, led to HBsAg loss in 24% of patients, with the mechanism involving activation of pDCs and HBV-specific CD8^+^ T cells (Lanford et al., 2013). Most studies on TLR7 agonists focus on the search for antivirals. For example, immunogenicity and safety of the TLR7 small molecule agonist SZU-101 (T7) coupled with inactivated H1N1 influenza virus vaccine were investigated in mice. In addition, the immuno-inducing activity of Flu-T7 on splenic lymphocytes was evaluated *in vitro*, and its inducing effect on specific antibodies was evaluated *in vivo*. It was demonstrated that Flu-T7 has good efficacy and safety, and is expected to be a new and efficient inactivated influenza vaccine (Zhang et al., 2014). In terms of its use as an adjuvant for antiparasitic vaccines, the TLR7 agonist R-837 can stimulate the activation of organismal neutrophils, produce reactive oxygen species (ROS), induce Th1-type cellular immunity, and enhance the immune effect of the Leishmania donovanii vaccine. On the other hand, another TLR7 agonist, imidazoquinolines, in combination with potent antimalarials, induced Th1-type cellular immunity and the production of IFN-*γ* and IL-12 to effectively control P. berghei infection in rodents (Regli et al., 2020; Saroa et al., 2019).

Major TLR8 agonists include VTXG233 (motolimod), GSG9688 (Selgantolimod), and TL8-506. The TLR8 agonist GS-9688 increases the frequency of MAIT cells in HBV patients and enhances their ability to produce IFN-γ (Amin et al., 2021). However, TLR7/8 agonists carry a relatively high risk of systemic inflammation. One study reported significant immune-related adverse events (irAEs) due to cytokine storm upon systemic administration, such as headache, fever, skin erosion, and myalgia, even exertional dyspnea, and potentially cytokine release syndrome (Ren et al., 2024). These risks somewhat limit their application for systemic administration. Amin et al. evaluated the potential of GS-9688 to promote viral control and modulate the response of regulatory mediators. GS-9688 enhanced antiviral effects through activation of a variety of immune cells, including HBV-specific CD8^+^ T cells, CD4^+^ follicular helper T cells, NK cells, and MAIT cells. GS-968 provides a potential biomarker and immunotherapeutic target (Amin et al., 2021). TLR8 and TLR7 can function in combination as vaccine adjuvants, and Soni et al. identified a very promising adjuvant in their study (Soni et al.). PVP-037, a member of the imidazopyrimidine family of molecules, targets the innate immune system to stimulate pattern recognition receptors such as TLR7 and TLR8 on antigen-presenting cells such as monocytes and dendritic cells. *In vivo*, PVP-037 enhanced the antibody response against influenza virus and SARS-CoV-2 vaccine proteins in mice, which provides a new candidate molecule and a direction for the development of new types of vaccine adjuvants (Soni et al., 2024).

### 4.3 TLR9: a “rising star” in clinical translation

TLR9 mainly recognizes unmethylated CpG DNA sequences, which are more common in bacteria and viruses but less frequent in mammals. Activation of TLR9 plays an important role in innate and adaptive immunity, especially in antiviral and antitumor immunity (Krieg, 2006). Upon recognition of CpG DNA, the intracellular TIR domain of TLR9 recruits MyD88, initiating the MyD88 signaling cascade. TLR9 activation also involves the TAK1-mediated activation of MAPK pathways like p38, JNK, and ERK, regulating cellular stress and inflammatory responses. In certain cell types, TLR9 activation involves IRFs and the induction of type I interferons (Huang and Yang, 2010).

TLR9 agonists have been investigated for their capacity to enhance vaccine efficacy and treat viral infections such as hepatitis B virus (HBV) and hepatitis C virus (HCV) infections, as well as emerging viruses such as SARS-CoV-2. For example, Yang et al. investigated the possibility of the TLR9 agonist CpG-ODN as a mucosal adjuvant for the SARS-CoV-2 mucosal vaccine (Yang et al., 2023). TLR9 agonists can also be used to stimulate the immune system’s ability to recognize and attack tumors. In a preclinical mouse tumor model, strong anti-tumor immunity was induced by subcutaneous vaccination with a long peptide together with CpGODN1826 and other adjuvants, clearing 80% of established HPV16 tumors (Huang et al., 2022). In another study, CpGODN1826 was reported to act as an adjuvant to a protein vaccine, inducing DC maturation and enhancing anti-tumor immune responses (Jie et al., 2018). The antitumor effects of CpGODN1826 and its use as an adjuvant in therapeutic oncology vaccines are currently at the animal experimental stage. Table 2 outlines the potential role of intracellular agonists.

**Table 2:** Potential role of intracellular agonists.

Agonist	Species	Role	References
Poly(I:C)	TLR3 agonist	Activates TLR3, induces the production of type I interferon and other cytokines, enhances antigen-specific CD8^+^ T-cell response, improves vaccine immunity, and enhances the anti-tumor effect of NK cells. Used in influenza, HIV, Zika virus, SARS-CoV-2 and other viral vaccine and tumor immunotherapy studies.	Salaun et al. (2006), Thomas et al. (2015), and Stowell et al. (2009)
NexAvant (NVT)	TLR3 agonist	Good stability as a promising adjuvant for antiviral or anticancer vaccines, overcoming the problems of Poly(I:C) as an adjuvant candidate.	Ko et al. (2023)
Imiquimod	TLR7 Agonist	Induces production of a variety of cytokines, activates immune cells, enhances the immune defense function, and exerts antiviral and anti-tumor effects. Used in the treatment of external genital and perianal condyloma acuminatum, superficial basal cell carcinoma and other diseases.	Skinner (2003)
GS-9620	TLR7 Agonist	By activating the TLR7 signaling pathway, GS-9620 induces the production of cytokines such as interferon and inhibits the replication of hepatitis B virus, and is being studied as a therapeutic option for chronic hepatitis B.	Lanford et al. (2013)
Flu-T7	TLR7 Agonist	Coupled with H1N1 influenza virus inactivated vaccine, Flu-T7 has good immunogenicity and safety and is expected to be a new and efficient inactivated influenza vaccine.	Zhang et al. (2014)
R-837	TLR7 Agonist	Stimulates the activation of neutrophils, generates reactive oxygen species (ROS), induces Th1-type cellular immunity, and enhances the immunological effect of *Leishmania* vaccines.	Regli et al. (2020)
Imidazoquinoline	TLR7 agonist	Combined with antimalarial drugs, it induces Th1-type cellular immunity, produces cytokines IFN-*γ* and IL-12, and effectively controls *P. berghei* infection in rodents.	Saroa et al. (2019)
GS-9688	TLR8 agonist	Activates multiple immune cells and enhances antiviral effects, providing potential biomarkers and immunotherapeutic targets for optimizing antiviral efficacy.	Amin et al. (2021)
PVP-037	TLR7/8 agonist	Enhances antibody responses against influenza virus and SARS-CoV-2 vaccine proteins, providing new candidate molecules and research directions for the development of novel vaccine adjuvants.	Soni et al. (2024)
CpG-ODN	TLR9 agonist	Used as a mucosal adjuvant for SARS-CoV-2 vaccine to induce DC maturation and enhance anti-tumor immune response for hepatitis B virus, hepatitis C virus and SARS-CoV-2 infections with enhanced therapeutic and vaccine effects.	Yang et al. (2023)
CpGODN1826	TLR9 agonist	Used as an adjuvant for protein vaccines to induce DC maturation and enhance anti-tumor immune response for therapeutic oncology vaccines.	Huang et al. (2022) and Jie et al. (2018)

## 5 Progress of TLR agonists as adjuvants for viral vaccines

Viral vaccines are an important means of preventing viral infections by mimicking the presence of a virus to activate the human immune system, thereby generating a protective immune response. The types of viral vaccines widely used include inactivated, live attenuated and polyvalent vaccines. Although vaccines against certain viruses exist, there are still many viruses for which there are no approved vaccines. In addition, treatment options for viral infections are limited due to the ineffectiveness of antibiotics against viruses, further highlighting the importance of viral vaccines for treatment and prevention. This underscores the critical need for adjuvants to develop safe and effective vaccines against viral infections (Pulendran et al., 2021).

There is increasing interest in the use of TLR agonists as immunomodulators to influence the outcome of treatment or prevention of diseases. Studies show that TLR agonists, as vaccine adjuvants and immunomodulators, can efficiently activate innate immune responses against infectious diseases and cancer through core signaling pathways like the MyD88-dependent pathway (detailed in Sections 2 and 3) (Luchner et al., 2021). The use of TLR agonists as vaccine adjuvants remains a focus of current research aimed at improving vaccine efficacy. This section explores the progress and availability of TLR agonists as vaccine adjuvants in different viral vaccines focusing on hepatitis B virus (HBV), SARS-CoV-2 hepatitis C virus (HCV), human immunodeficiency virus (HIV), influenza virus, and flaviviruses. Table 3 outlines the roles of various TLR adjuvants in viral vaccines.

**Table 3:** TLRs as a vaccine adjuvant for viral vaccines.

Virus	DNA/RNA	Targeted TLR	Primary agonists	References
HBV	DNA	TLR4	MPLA	Tran (2011), Sukriti et al. (2010), Wang et al. (2014), Daffis et al. (2021), Lee and Lim (2021), Eng et al. (2013), Kundi (2007), Whitaker et al. (2012), and Marks et al. (2023)
		TLR7/8	CL097 GS-9688	
		TLR9	CpG 1018	
HCV	RNA	TLR3	50s ribosomal protein L7/L12	Petta and Craxì (2020), Behmard et al. (2022), McHutchison et al. (2007), and Dominguez-Molina et al. (2018)
		TLR4	Human β-defensin 2	
		TLR7	AT2	
		TLR9	CpG 10101	
HIV	RNA	TLR4	MPLA (SMNP)	Zhao et al. (2025), Anang et al. (2025), Williamson et al. (2025), Wille-Reece et al. (2005), Moody et al. (2014), Anguille et al. (2015), Zhu et al. (2010), Gutjahr et al. (2020), Kasturi et al. (2020), and Silva et al. (2021)
		TLR7/8	3 M-012 NOD2L 3 M-052	
SARS-CoV-2	RNA	TLR2/6	INNA-051	Lasrado et al. (2025), Abbad et al. (2025), Proud et al. (2021), Atalis et al. (2022), Abhyankar et al. (2021), and Zhang et al. (2022)
		TLR4	GLA Lipid A (MPLA)	
		TLR7/8	3 M-052 R848 Gardiquimod	
Influenza virus	RNA	TLR2	Pam2Cys	Maurer et al. (2025), Abozeid et al. (2025), Xu et al. (2021), Hussell and Goenka (2016), Hajam et al. (2017), Liu et al. (2011), Goff et al. (2015), Sato-Kaneko et al. (2020), Kaushik et al. (2020), Jangra et al. (2022), and Clemens et al. (2022)
		TLR4	1Z105	
		TLR5	Flagellin	
		TLR7/8	BBIQ Imidazoquinoline R848 1 V270	
Flavivirus	RNA	TLR4	SLA MPLA	Subramaniam et al. (2020), Bhatt et al. (2013), Beltramello et al. (2010), Bardina et al. (2017), Van Hoeven et al. (2018), McDonald et al. (2007), Kayesh et al. (2021), Espinosa et al. (2019), Schneider et al. (2023), and Gosavi and Patil (2022)
		TLR5	Flagellin	

### 5.1 TLR agonists as vaccine adjuvants in HBV vaccines

The core immunological barrier in chronic HBV infection is profound immune tolerance, primarily manifested as weak HBV-specific T cell responses and potentially impaired antigen processing and presentation via MHC class I molecules. This state of tolerance significantly limits the efficacy of traditional vaccines, urgently necessitating adjuvants that can reverse tolerance and rebuild potent antiviral immunity (Tran, 2011; Sukriti et al., 2010).

In studies to overcome HBsAg-specific immune tolerance in a humanized mouse model, HBV-Ag immunization coupled with CL097 (TLR7/8 agonist) reversed immune tolerance and induced antigen-specific immune responses in HBV-Transgenic mice. TLR7/8 agonists have demonstrated potent adjuvant properties in the induction of antigen-specific Th1 responses to overcome immune tolerance (Wang et al., 2014). A different study reported that GS-9688 induced cytokines in human peripheral blood mononuclear cells that activated antiviral effector functions by increasing the frequency of HBV-specific CD8^+^ T cells, CD4^+^ follicular helper T cells, NK cells, and mucosal-associated invariant T cells (Daffis et al., 2021). HEPLISAV-B, a recombinant HBV vaccine consisting of HBsAg in combination with a CpG 1018 adjuvant contributed to the stimulation of innate immunity through TLR9 (Lee and Lim, 2021). In phase III clinical trials, HEPLISAV-B rapidly and consistently induced high titers of sustained seroprotection with fewer immunizations, including in individuals who responded poorly to vaccination (Eng et al., 2013). FENDrix, a vaccine currently used to protect patients with kidney problems against hepatitis B, is an adjuvanted HBV vaccine consisting of recombinant HBsAg formulated with aluminum phosphate and MPLA. FENDrix is administered in a four-dose regimen: day 0, month 1, month 2, and month 6 (after day 0) which induces higher concentrations of protective antibodies in a shorter period of time (Kundi, 2007).

**Figure 3:**
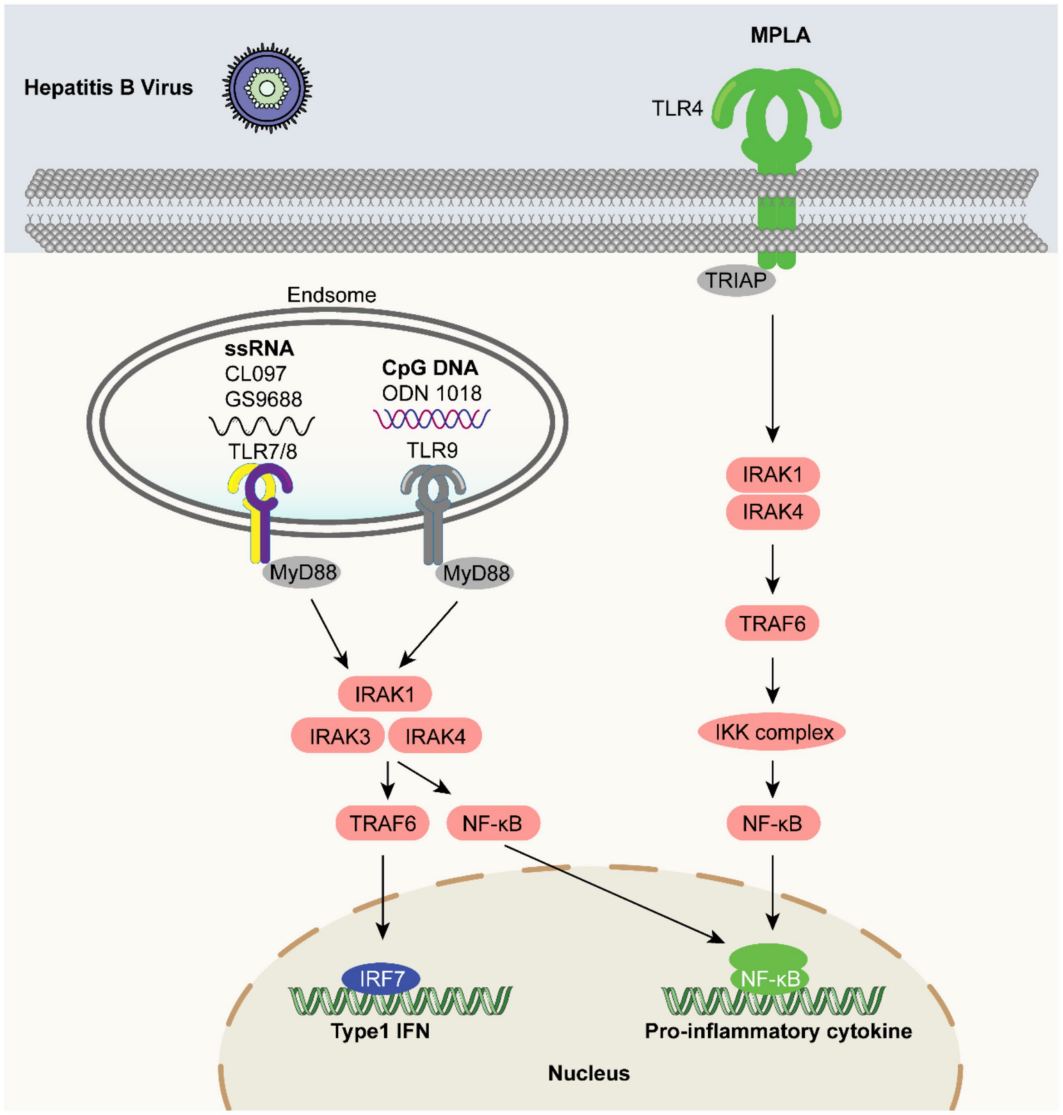
TLR agonists as vaccine adjuvants in HBV vaccines. The small molecule agonist MPLA activates the TLR4 receptor. The natural ligand of TLR7 and TLR8 is ssRNA. Small molecule IMQ compounds such as CL097 and GS-9688 also activate both receptors and are the subject of current adjuvant research.

HIV/HBV co-infections may result in increased morbidity and mortality compared to HBV or HIV mono-infections and new options are needed to enhance the HBV vaccine response in immunocompromised individuals (Whitaker et al., 2012). A recent study investigated the use of HEPLISAV-B vaccine adjuvanted with the TLR9 agonist adjuvant in HIV-positive individuals who had not previously received an HBV vaccine. The study findings noted that all 68 participants achieved HBV seroprotective titers after 3 doses, with no other safety concerns (Marks et al., 2023). These findings highlight the increased immunogenicity of the HBV vaccine and the potential of TLR agonists as immunomodulators in enhancing the efficacy of vaccination (Figure 3).

### 5.2 TLR agonists as vaccine adjuvants in HCV vaccines

Hepatitis C Virus (HCV) can cause chronic liver infection. Its long-term harm primarily lies in inducing liver cancer. The key to clearing chronic HCV infection and preventing hepatocellular carcinoma is inducing potent and durable antigen-specific T cell immunity—this is the core breakthrough direction for current HCV vaccine development (Petta and Craxì, 2020). There is currently no approved preventive HCV vaccine. The development of an effective vaccine with the core goal of “inducing strong T cell immunity” is urgently needed. TLR agonists, leveraging their ability to activate innate immunity and amplify adaptive immune responses, have become important tools to achieve this goal.

**Figure 4:**
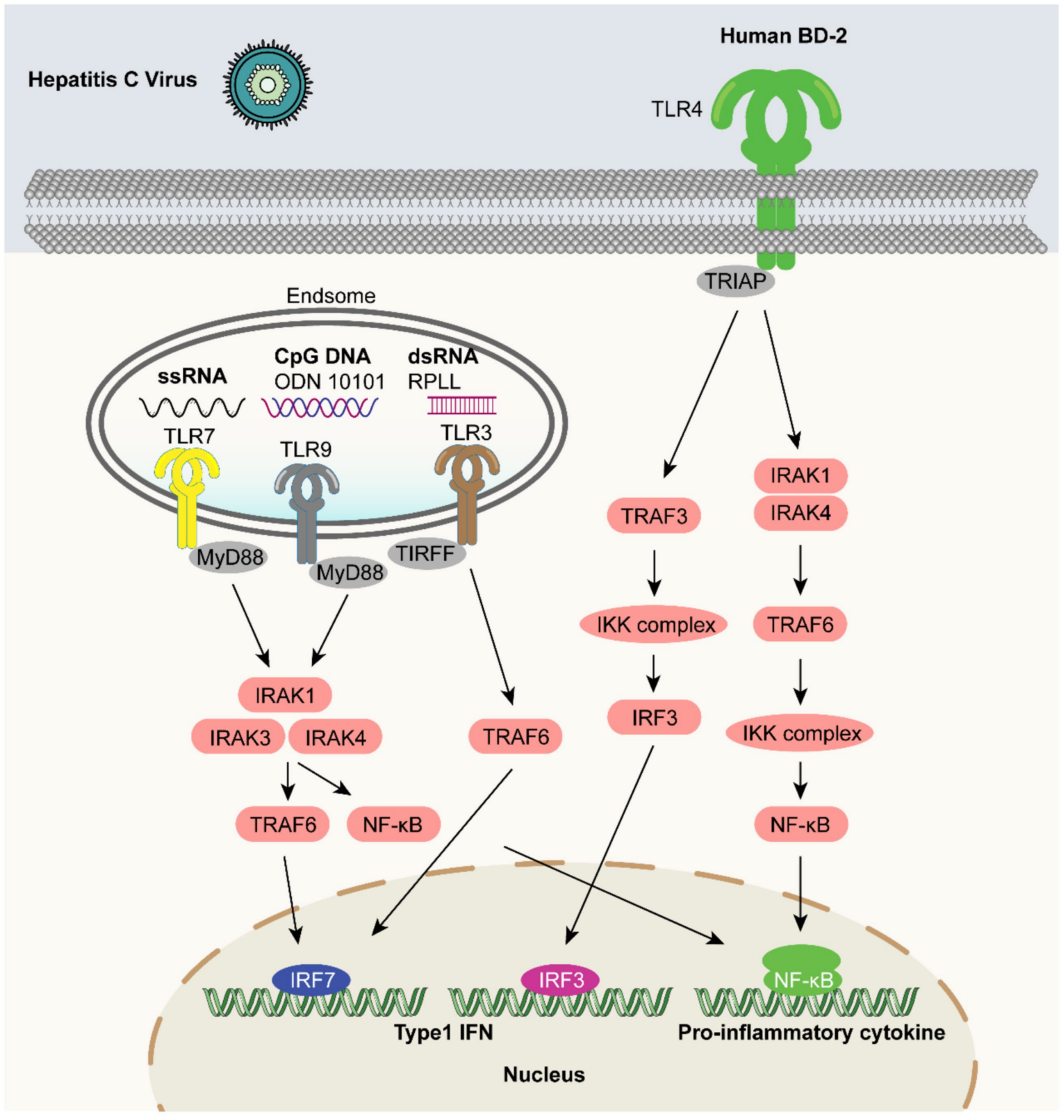
TLR agonists as vaccine adjuvants in HCV vaccines. Human *β*-defensin 2 can act as a ligand to bind to the TLR4 receptor, recruit the adaptor protein TRIF, and then activate TRAF3. TRAF3 triggers the IKK complex, causing IRF3 to be phosphorylated and transported into the cell nucleus, where it participates in the regulation of immune-related genes. ssRNA is the natural ligand of TLR7, and dsRNA can activate TLR3. After activation, both transduce signals through TRIF, and the activation of TLR3 also involves the 50S ribosomal proteins L7/L12. CpG RNA can serve as a ligand for TLR9. TLR9 agonists such as CpG10 and CpG101 first bind to the MyD88 adaptor protein, sequentially activate IRAK1, IRAK4, and IRAK3, and then via TRAF6, promote the activation of NF-κB and its translocation into the cell nucleus. The above signaling pathways mediated by the TLR family will ultimately induce the production of pro-inflammatory cytokines and type I interferons (Type 1 IFNs) in the cell nucleus, play a crucial role in the process of innate immune defense, and provide an important molecular pathway basis for the research on antiviral immune mechanisms, as well as the development of related drugs and vaccine adjuvants.

The use of immunoinformatic-based multi-epitope constructs in combination with TLR3 and TLR4 agonists, has demonstrated immunogenic, non-allergenic, and non-toxic properties, and further investigations on the protective properties and safety of these design candidates is needed. Several studies have reported improved efficacy of HCV vaccine candidates that contain TLR agonists (Behmard et al., 2022). In one study, the TLR9 agonist CpG 10101 increased the activation of immune markers while decreasing HCV RNA levels, in a dose-dependent manner (McHutchison et al., 2007). These findings proposed that CpG 10101 could be trialed as an adjuvant for HCV vaccine candidates. Furthermore, the addition of TLR7 and TLR9 agonists to HCV vaccine candidates has been reported to promote the maturation of plasma cell-like dendritic cells, thereby improving antigen presentation and enhancing viral immunity (Dominguez-Molina et al., 2018) (Figure 4).

### 5.3 TLR agonists as vaccine adjuvants in HIV vaccines

HIV infection presents unique immunological challenges: firstly, the virus forms an “immunological shield” through the high glycosylation of its envelope protein (Env), while utilizing frequent genetic recombination and mutation, making it difficult for the host to produce broadly neutralizing antibodies against different strains; secondly, the virus can establish latent reservoirs, where latent cells do not express viral antigens, evading immune recognition and clearance, and can rapidly activate and release virus upon cessation of therapy (Zhao et al., 2025; Anang et al., 2025; Williamson et al., 2025). Therefore, developing an HIV vaccine capable of generating high-titer broadly neutralizing antibodies and clearing viral reservoirs remains a major focus, and research is ongoing into the single or combined use of TLR agonists to develop TLR-agonist-adjuvanted HIV vaccines.

**Figure 5:**
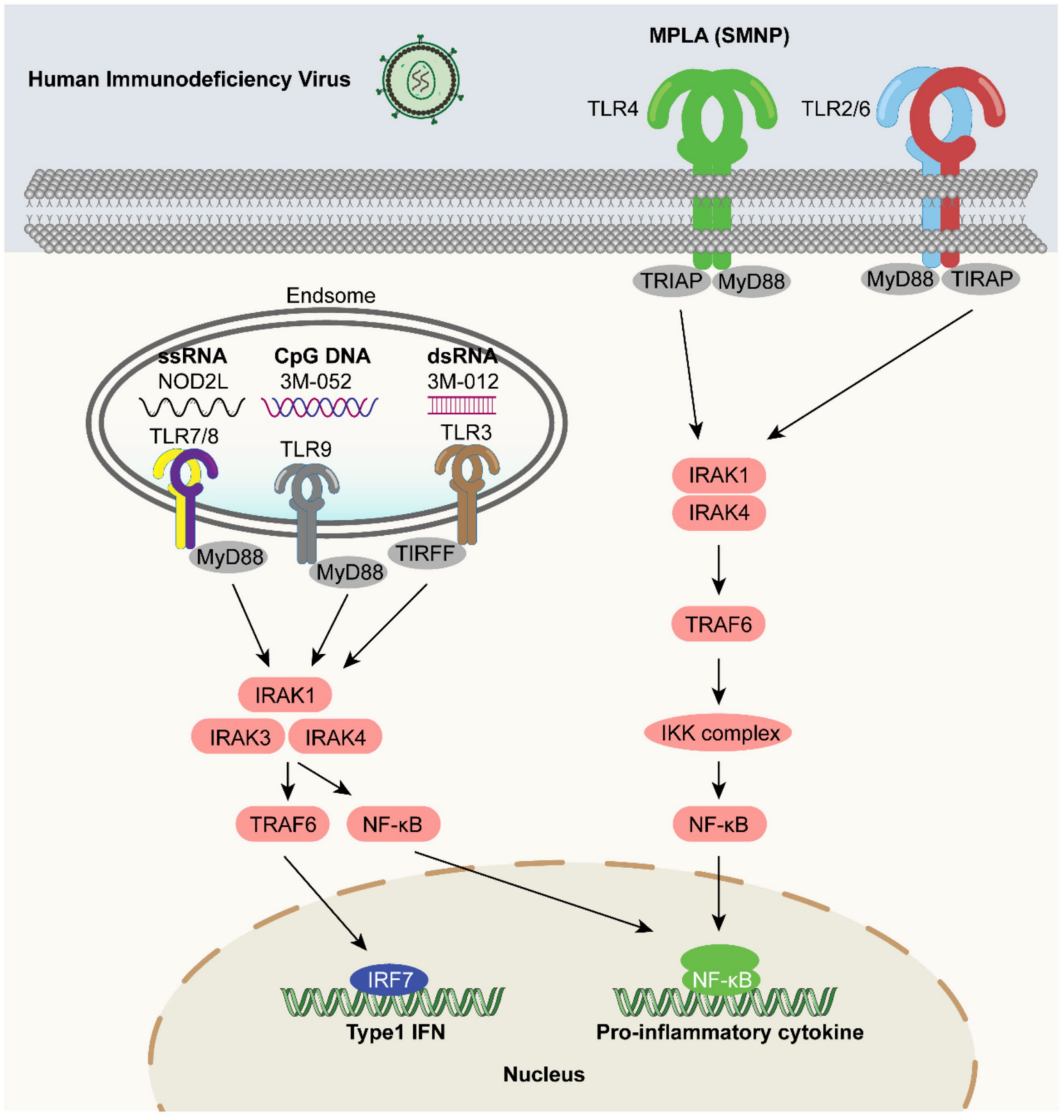
TLR agonists as vaccine adjuvants in HIV vaccines. TLR agonists play a significant role in immune-signaling pathways. MPLA (SMNP) activates TLR4 on the cell surface, and through a series of steps involving TIRAP, MyD88, IRAK1, IRAK4, TRAF6, and the IKK complex, ultimately leads to NF-κB triggering the production of pro-inflammatory cytokines. TLR7 and TLR8, located in endosomes, can be activated by ssRNA and small-molecule IMQ compounds such as CL097 and GS-9688. This activation proceeds through MyD88, IRAK1, IRAK4, IRAK3, TRAF6, and NF-κB, promoting the production of type 1 IFNs and pro-inflammatory cytokines. TLR3 in endosomes is activated by dsRNA, and through TIRF, IRAK1, IRAK4, TRAF6, and NF-κB, cytokines are produced. TLR9 in endosomes is activated by CpG RNA, and through MyD88, IRAK1, IRAK4, IRAK3, TRAF6, and NF-κB, it participates in the immune response. These pathways are of great importance for adjuvant research.

Wille et al. have shown that HIV Gag proteins coupled to a TLR7/8 agonist (3 M-012) enhanced the intensity of Th1 and CD8^+^ T cell responses in non-human primates (Wille-Reece et al., 2005). In a rhesus monkey model, TLR agonists increased the level of epitope-specific HIV-1 Env-reactive antibodies, and the combination of TLR7/8 and TLR9 agonists triggered higher titers of neutralizing and antibody-dependent cell-mediated cytotoxicity -mediated antibodies (Moody et al., 2014). Ligands combining three TLRs (TLR2/6, TLR3, and TLR9) increased DC and IL-15 production as well as promoted DC activation and NK cell stimulation (Anguille et al., 2015). This combination also greatly enhanced the protective efficacy of HIV envelope peptide vaccines in a mouse model (Zhu et al., 2010). Gutjahr et al. demonstrated that intranasal administration of TLR7/NOD2L agonists with the NP-p24 HIV vaccine produced an effective adjuvant effect, inducing high-quality humoral and adaptive immune responses in both systemic and mucosal compartments (Gutjahr et al., 2020). 3 M-052 is a TLR7 and TLR8 agonist adjuvant that induces a significantly high and sustained frequency of Env-specific long-lived plasma cells in bone marrow (up to ~1 year) and serum antibody responses in Rhesus monkeys (Kasturi et al., 2020). Recent studies have shown that nanoparticle adjuvants based on TLR4 agonists, saponin/MPLA nanoparticles (SMNPs), enhance lymphatic blood flow and antigen entry into lymph nodes in animal models. A single dose of vaccination using an Env trimer in combination with SMNP adjuvant resulted in seroconversion of all vaccinated male and female Indian Rhesus monkeys with high HIV-neutralizing antibody titers (Silva et al., 2021). This suggests that SMNP is a promising vaccine adjuvant candidate for clinical applications against HIV infection (Figure 5).

### 5.4 TLR agonists as vaccine adjuvants in the SARS-CoV-2 vaccine

Respiratory viral infections (like SARS-CoV-2) remain a major threat to global human health. Their unique immunological challenges are reflected in the “weak mucosal immune defense and immune escape by variants”: SARS-CoV-2 primarily invades through the respiratory mucosa, but the body’s local mucosal immune response against respiratory viruses is short-lived. Furthermore, the viral spike protein (S protein) is prone to amino acid mutations (e.g., over 30 mutation sites in the Omicron variant), leading to reduced binding capacity of existing neutralizing antibodies and resulting in immune escape (Lasrado et al., 2025). Although global COVID-19 vaccination programs have been successful, issues such as short duration of immunity, insufficient effectiveness against new variants, difficulty in preventing infection and transmission, and low global accessibility need urgent addressing. Strengthening mucosal immunity and enhancing cross-protection against variants are core directions for optimizing COVID-19 vaccines (Abbad et al., 2025). TLR agonists offer new ideas for solving these problems.

It has been shown that intranasal administration of the TLR2/6 agonist INNA-051 in a ferret model significantly reduced SARS-CoV-2 viral RNA levels in the nose and throat (Proud et al., 2021). In addition, SARS-CoV-2 subunit vaccines supplemented with TLR4 and RIG-I agonists have been shown to induce robust and unique pathway-specific adaptive immune responses against SARS-CoV-2 (Atalis et al., 2022). SARS-CoV-2 spike subunit vaccines adjuvanted with dual TLR ligand liposomes induced potent systemic neutralization in a COVID-19 mouse model and complete protection against lethal SARS-CoV-2 attack (Abhyankar et al., 2021). These findings highlight the need for further studies to identify ideal adjuvants for SARS-CoV-2 vaccines.

**Figure 6:**
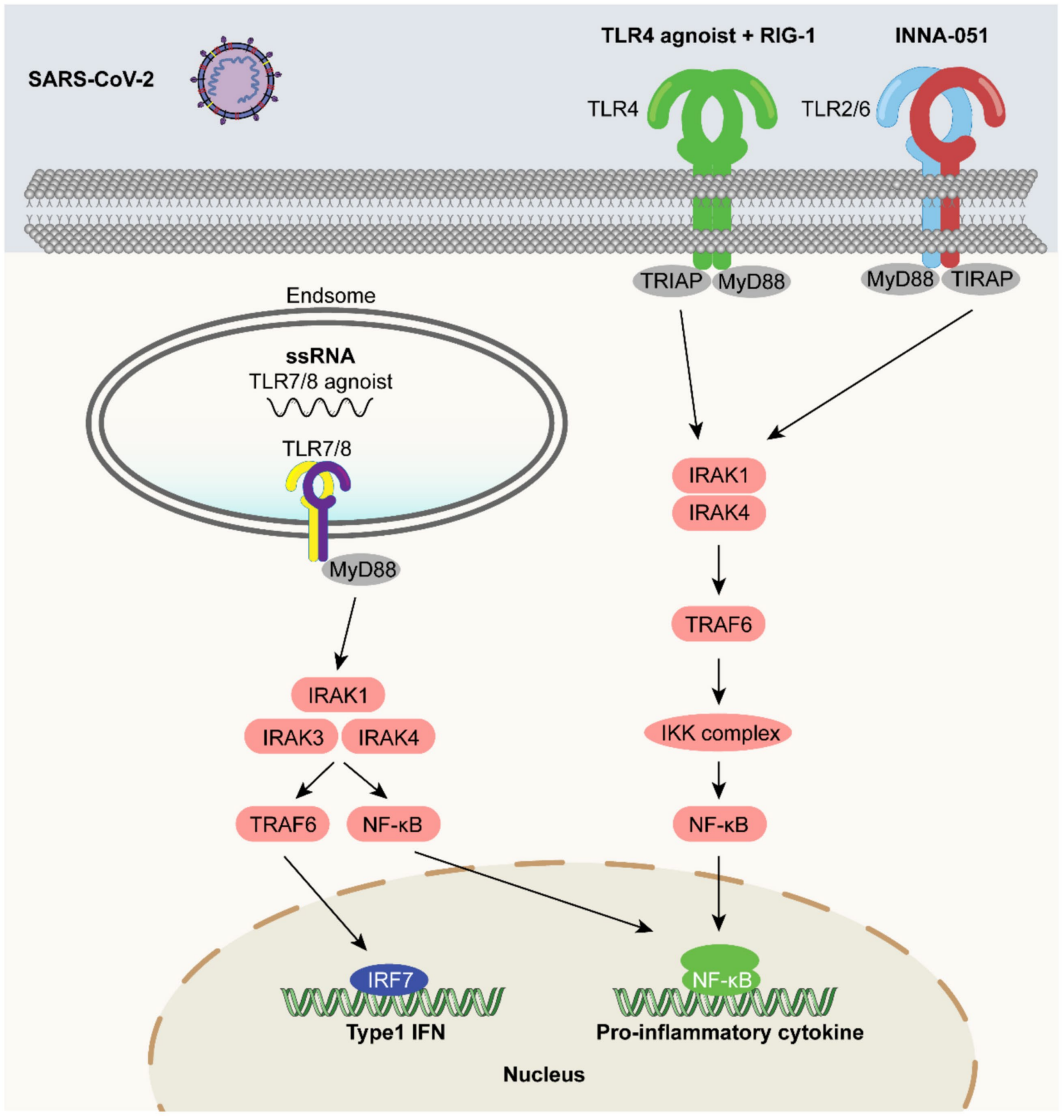
TLR agonists as vaccine adjuvants in the SARS-CoV-2 vaccine. SARS-CoV-2-related molecular patterns trigger TLR-mediated signaling: TLR4 agonist + RIG-1 and INNA-051 bind to membrane-bound TLR4, TLR2/TLR6, recruiting TIRAP and MyD88 to activate IRAK1, IRAK4, and then TRAF6, which activates the IKK complex for NF-κB activation and nuclear entry; ssRNA (or its agonist) activates endosomal TLR7/8, with recruited MyD88 triggering IRAK1, IRAK4, IRAK3, then TRAF6 to activate NF-κB for nuclear entry. These pathways induce pro-inflammatory cytokines and Type 1 IFNs in the nucleus, playing key roles in innate immunity against SARS-CoV-2 and providing a basis for related research and development.

Recent reports have highlighted the efficacy of TLR7 agonist-adjuvanted vaccines in inducing robust immune responses against SARS-CoV-2. Inoculation using the S1 subunit of the SARS-CoV-2 spike protein, supplemented with a TLR7 agonist, induced effective humoral and cellular immunity in mice. This approach produced a balanced Th1/Th2 immune response and efficiently induced neutralizing antibodies against SARS-CoV-2 and all variants of interest (B.1.1.7/alpha, B.1.351/beta, P.1/gamma, B.1.617.2/delta, and B.1.1.529/omicron), demonstrating great potential for this adjuvant-protein coupled vaccine candidate (Zhang et al., 2022). These findings reiterate the promise of TLR agonists as potential adjuvants (Figure 6).

### 5.5 TLR agonists as vaccine adjuvants in influenza virus vaccines

Influenza viruses are prone to antigenic drift and shift. Their unique immunological challenges manifest as “narrow immune memory and the risk of Antibody-Dependent Enhancement (ADE)”: traditional influenza vaccines often induce immune responses targeting the variable head region of the viral hemagglutinin (HA), which only recognizes specific strains and cannot cope with new variants generated by antigenic drift; some influenza viruses (like H5N1) may also cause ADE, where non-neutralizing antibodies bind to the virus and instead facilitate its entry into host cells, exacerbating infection (Maurer et al., 2025; Abozeid et al., 2025; Xu et al., 2021). Therefore, developing highly effective vaccines that achieve cross-protection and rapidly induce immune responses is key to combating influenza virus infections and remains an important goal of modern medical research. Studies to improve influenza virus vaccines with TLR agonists are under development. A reduction in influenza-associated secondary pneumococcal infections has been reported in mice co-administered with inhaled TLR2 agonists and inactivated vaccines, highlighting the effectiveness of TLR agonists in influenza vaccines (Hussell and Goenka, 2016). TLR5 agonists elicit the induction of cytokines and chemokines, and also show the potential for the activation of immune cells, and the initiation of both innate and adaptive immune responses. The use of bacterial flagellins as TLR5 agonists in vaccines appears to show promise (Hajam et al., 2017). Notably, flagellin has been extensively studied as a mucosal adjuvant in epitope-based influenza vaccines (Liu et al., 2011).

**Figure 7:**
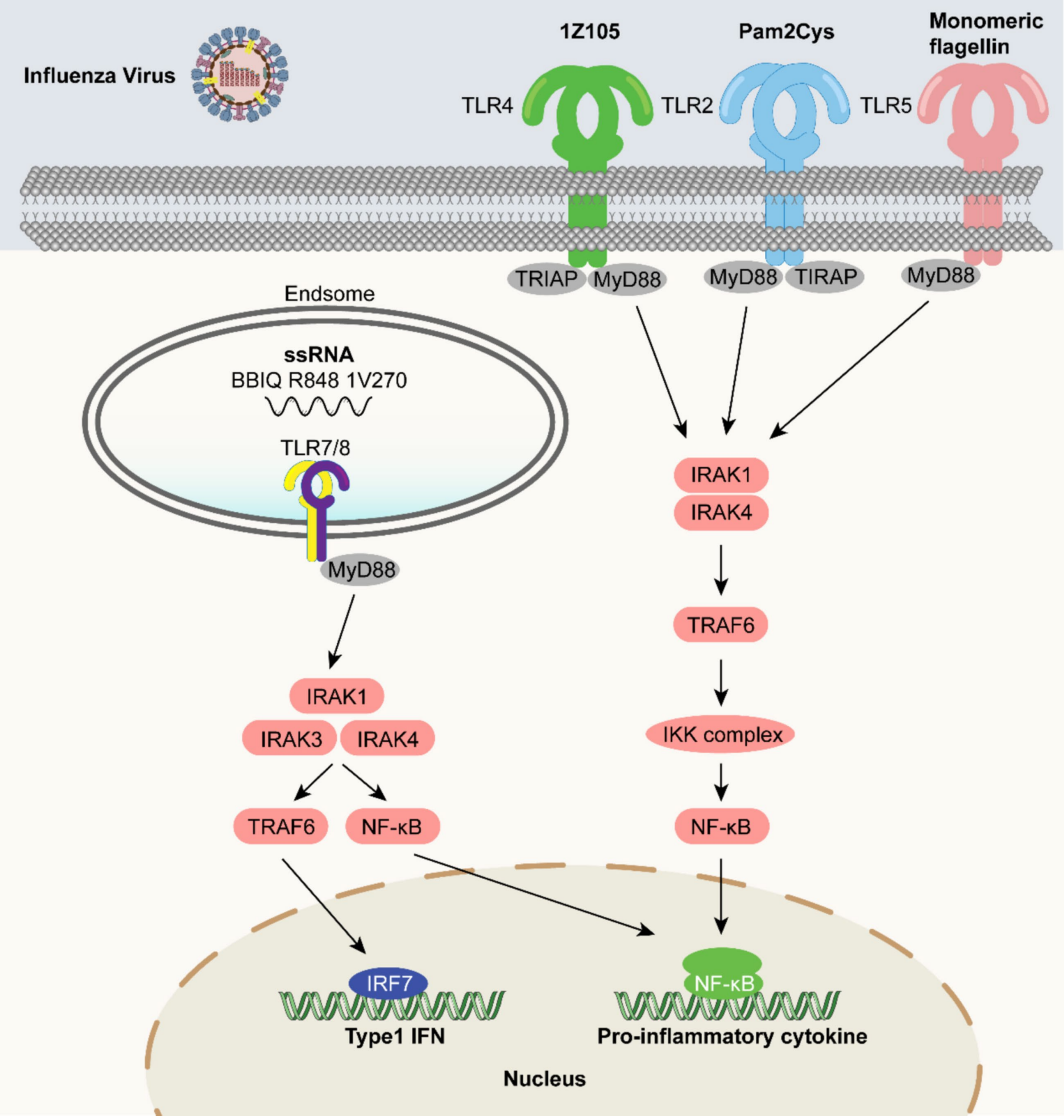
TLR agonists as vaccine adjuvants in influenza virus vaccines. Influenza virus-related molecular patterns activate TLRs (TLR4 by 12105, TLR2 by Pam2Cys, TLR5 by flagellin, and TLR7/8 by ssRNA/agonists like BB1Q). These TLRs recruit adaptor proteins (TIRAP/MyD88 for TLR4/2, MyD88 for TLR5/TLR7/8), activating IRAK1/4, then TRAF6 and the IKK complex. This leads to NF-κB activation and nuclear translocation, inducing pro-inflammatory cytokines and Type 1 IFNs production, playing key roles in anti-influenza innate immunity and providing a basis for related research and development.

In a mouse model, Goff et al. reported that the combination of recombinant haemagglutinin (HA) from the influenza A virus strain A/Puerto Rico/8/1934 (rPR/8HA) with TLR4 and TLR7 ligands induced rapid and sustained humoral immunity against a lethal attack by viruses with homologous HA (Goff et al., 2015). Another study showed that the dual combination of TLR4 and TLR7 ligands in recombinant influenza virus HA vaccines induced a broader immune response (Sato-Kaneko et al., 2020). Among the imidazoquinolinone compounds, 1-benzyl-2-butyl-1H-imidazo[4,5-c]quinolin-4-amine (BBIQ) is a potential TLR7 agonist, and it was demonstrated that a recombinant influenza HA protein vaccine administered with BBIQ significantly enhanced anti-influenza IgG1 and IgG2c responses in mice (Kaushik et al., 2020). In another study, a licensed quadrivalent inactivated influenza vaccine (QIV) administered with RIG-I (SDI-nanogel) and a TLR7/8 agonist (imidazoquinolinone) enhanced antibody and T-cell responses associated with protection against lethal influenza virus infections (Jangra et al., 2022). Clemens et al. demonstrated that vaccination with a TLR7/8 agonist (R848) in combination with influenza A virus vaccine elicited antibody responses to the highly conserved hemagglutinin stem and promoted rapid induction of virus neutralizing stem-specific antibodies upon challenge with the A/Puerto Rico/8/1934 (H1N1) strain (Clemens et al., 2022). Figure 7 summarizes how the use of TLR agonists in influenza vaccines could contribute to the development of more effective vaccines.

### 5.6 TLR agonists as vaccine adjuvants in flavivirus vaccines

The Flavivirus family (including West Nile virus, Dengue virus, Chikungunya virus, etc.) presents unique immunological challenges centered on “serotype cross-interference and Antibody-Dependent Enhancement (ADE)”: Flaviviruses share common antigenic epitopes among serotypes. Antibodies produced after infection with one serotype may bind to viruses of other serotypes but fail to neutralize them; instead, they can facilitate virus entry into target cells like macrophages via Fc receptor mediation, enhancing the risk of infection (Subramaniam et al., 2020; Bhatt et al., 2013; Beltramello et al., 2010; Bardina et al., 2017). This immunological challenge means that vaccines against a single serotype might increase the risk of infection with other serotypes. Therefore, developing vaccines that achieve cross-serotype protection is a core need, and TLR agonists play an important auxiliary role in this endeavor.

**Figure 8:**
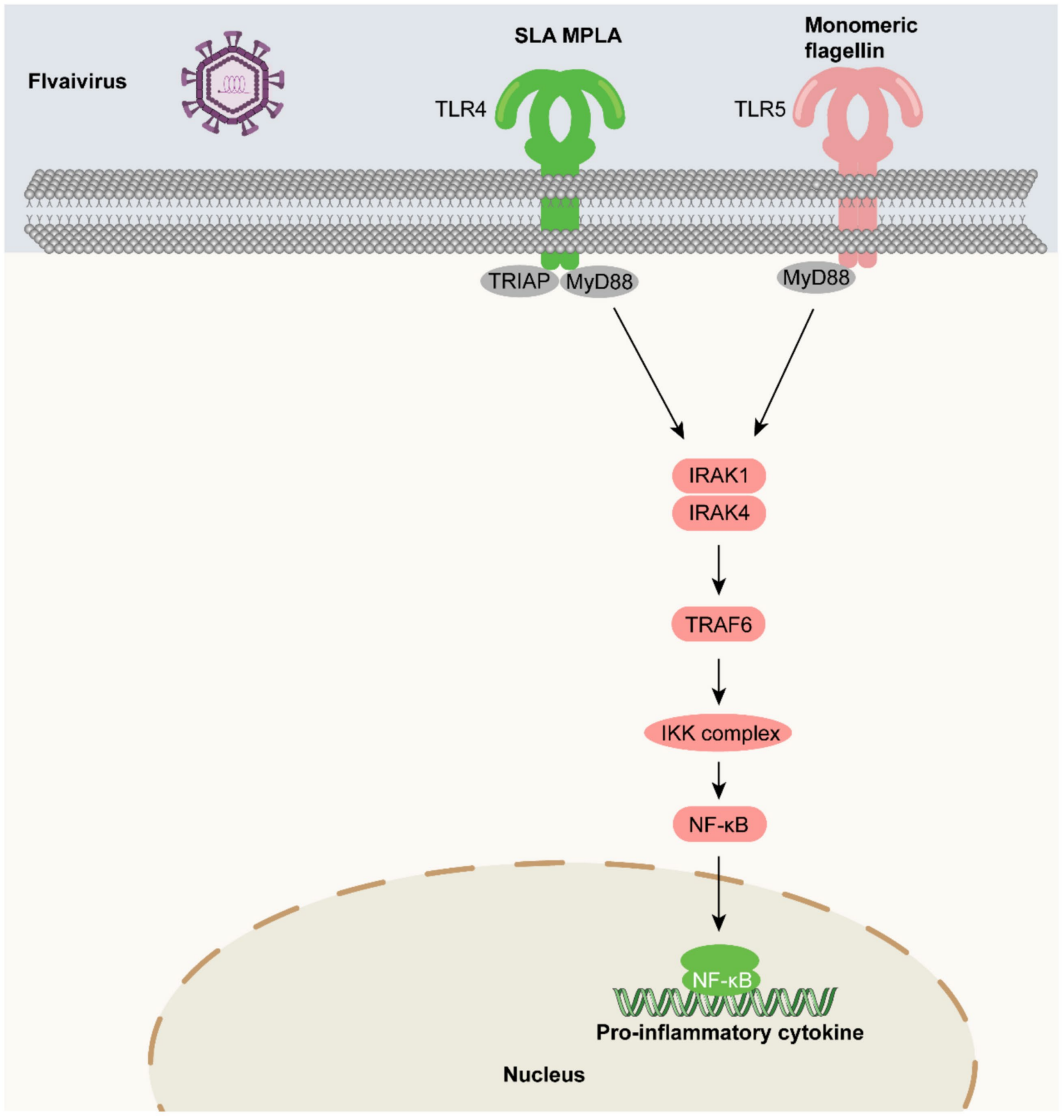
TLR agonists as vaccine adjuvants in flavivirus vaccines. Flavivirus-related molecular patterns trigger TLR-mediated signaling: TLR4 agonist SLA MPLA binds to membrane-bound TLR4, recruiting TIRAP and MyD88, and TLR5 agonist (flagellin) binds to TLR5, recruiting MyD88. Both activate IRAK1 and IRAK4, then TRAF6, which activates the IKK complex for NF-κB activation and nuclear entry. These pathways induce pro-inflammatory cytokines in the nucleus, playing key roles in innate immunity against flavivirus and providing a basis for related research and development.

There is currently no approved human vaccine for West Nile virus (WNV). In a recent study, a WNV recombinant E-protein vaccine (WN-80E) adjuvanted with the TLR4 agonist SLA (second-generation lipid adjuvant) or the saponin adjuvant QS21 was able to induce a long-lasting immune response in a preclinical model that successfully reduced the WNV viral titer (Van Hoeven et al., 2018). Flagellin has also been investigated as a mucosal adjuvant for use in WNV recombinant protein vaccines to induce a protective immune response (McDonald et al., 2007). There is an urgent need for a DENV vaccine that is equally effective against all four serotypes of the dengue virus. For the development of a pan-serotype dengue vaccine, TLR agonists may prove useful as adjuvants (Kayesh et al., 2021). A recent study investigated the immunogenicity and protective capacity of recombinant DENV NS1 administered with CDN. It was observed that NS1-CDN immunization induced serotype-specific and cross-reactive antibodies and T-cell responses in a mouse model. In addition, NS1-CDN vaccination protected against homotypic and heterotypic DENV-2-induced morbidity and mortality (Espinosa et al., 2019). Until recently, there was no clinically approved chikungunya virus (CHIKV) vaccine for immunization; however, in November 2023, the US Food and Drug Administration approved the first chikungunya vaccine, Ixchiq/VLA1553 (for use in individuals 18 years of age and older at increased risk of CHIKV exposure (US Food and Drug Administration, 2023)). In a multicenter, randomized, placebo-controlled phase 3 clinical study, the CHIKV vaccine VLA1553 was established to be generally safe and well tolerated in young and older adults, inducing seroprotective chikungunya virus neutralizing antibody levels in 98.9% of participants (Schneider et al., 2023). A recent study reported enhanced efficacy of the inactivated CHIKV-MPLA combination, which induced higher levels of neutralizing antibodies compared to the unadjuvanted CHIKV vaccine. Although further studies are needed, TLR4 agonists are a promising adjuvant candidate to enhance the efficacy of CHIKV vaccines (Gosavi and Patil, 2022) (Figure 8).

### 5.7 Comparative analysis of TLR agonist applications across viral vaccines

The strategy for selecting TLR agonists for different viral vaccines stems primarily from the unique immune challenges posed by each viral infection: HBV involves deep immune tolerance, requiring TLR7/8/9 agonists to activate the MyD88-dependent pathway, reverse T cell hyporesponsiveness, and rebuild antiviral immunity; respiratory viruses (Influenza, SARS-CoV-2) primarily spread via mucosal infection, thus favoring TLR2/4/5 agonists to strengthen the mucosal immune barrier and block infection and transmission; HIV requires the simultaneous induction of broadly neutralizing antibodies and potent cellular immunity to clear latent reservoirs, hence often employing combinations like TLR4 with TLR7/8 agonists to synergistically activate both humoral and cellular immunity; For Hepatitis C Virus (HCV), whose primary pathologies are chronic infection and induction of hepatocellular carcinoma, the requirement is for TLR3/4/9 agonists to enhance antigen presentation efficiency and induce potent T cell immunity for viral clearance. In contrast, flaviviruses face the challenge of serotype cross-interference, necessitating reliance on TLR4 agonists or multi-target TLR combinations to induce cross-reactive serotype immune responses and achieve pan-serotypic protection.

## 6 Clinical translation status and challenges of TLR agonists

### 6.1 Overview of approved and clinically investigated TLR agonists

Currently, four TLR agonists have been approved globally for use as vaccine adjuvants. This review summarizes TLR agonists that have entered clinical stages in the past 5 years (Table 4).

**Table 4:** TLR agonists that have entered clinical stages in the past 5 years.

Target drug	Name	Number	Status	Phase	Key results	References
TLR3	Rintatolimod	NCT00215800	Completed	Phase III	For Myalgic Encephalomyelitis/Chronic Fatigue Syndrome (ME/CFS): In patients with a symptom duration of 2–8 years, 51.2% achieved a clinically meaningful improvement of ≥25% in exercise duration.	Strayer et al. (2020)
TLR4	NI-0101	NCT03241108	Completed	Phase II	For Rheumatoid Arthritis (RA): A Phase II clinical trial of a monoclonal antibody targeting this indication has been completed; however, the results have not yet been disclosed.	Xu et al. (2025) and Tam et al. (2021)
TLR7	RO7020531	NCT04225715	Completed	Phase I	For Chronic Hepatitis B Virus (HBV) Infection: Administration of 150 mg or 170 mg doses every other day resulted in detectable IFN-*α* levels in all patients and a significant reduction in HBV DNA concentration. The regimen demonstrated a favorable safety profile and was well-tolerated.	Yuen et al. (2023)
TLR7/8	AD7/8	Preclinical	Preclinical	N/A	As a Novel TLR7/8 Agonist: It was incorporated into lipid nanoparticles (LNP) to serve as an adjuvant for mRNA vaccines. Preclinical studies demonstrated a significant enhancement of antigen-specific T-cell and antibody responses.	Choi et al. (2025)
TLR9	Cavrotolimod (AST-008)	NCT03086278	Completed	Phase I	First-in-Human Trial in Healthy Participants: Results indicated that it was safe and well-tolerated, with no dose-limiting toxicities observed. It effectively activated innate immunity and elicited a Th1-type immune response.	Daniel et al. (2022)
TLR9	CpG7909	Preclinical	Preclinical	N/A	Administered via intravenous infusion in combination with anti-PD1 antibody: In animal models, this combination therapy effectively converted the tumor microenvironment from an immunologically “cold” to “hot” phenotype, markedly promoting CD8^+^ T cell infiltration and inducing durable tumor regression.	Tu et al. (2025)

Data source: ClinicalTrials.gov. Data retrieval period: January 2020 – November 2025; All studies are publicly searchable ongoing or completed phase-specific trials.

### 6.2 Core clinical trial data: immunogenicity and safety

The core of clinical translation for TLR agonists lies in balancing their potent immunostimulatory capacity with the resulting inflammatory toxicity. Regarding immunogenicity, their success is reflected in the effective activation of innate immunity (manifested as transient increases in cytokines like IFN-*α*, IL-6, IP-10 and interferon-stimulated genes in peripheral blood post-administration) and successful bridging to adaptive immunity, i.e., significantly enhancing antigen-specific antibody titers (and modulating IgG subclasses to bias Th1/Th2 responses) when used as vaccine adjuvants, or activating and recruiting tumor antigen-specific CD8^+^ T cells to the tumor microenvironment upon intratumoral injection (e.g., Tilsotolimod and CMP-001) (Haymaker et al., 2021; Milhem et al., 2025). However, their mechanism of action directly leads to the main safety challenges: the most common adverse events are injection site reactions and reversible flu-like symptoms (fever, chills, fatigue), while at higher doses, substantial cytokine release may cause cytokine release syndrome (Doebele et al., 2020). Therefore, modern clinical strategies emphasize finding the optimal biologically effective dose rather than the maximum tolerated dose. Additionally, specific risks exist for different targets; for instance, systemic TLR4 agonists may induce sepsis-like reactions, while TLR7/8/9 agonists require attention to their potential risk of inducing autoimmune-like symptoms (Hug et al., 2020; Del Prete et al., 2019).

### 6.3 Translation bottlenecks

The clinical translation of TLR agonists primarily faces three major bottlenecks:

a) Inflammatory toxicity: The primary dose-limiting toxicity of TLR agonists stems from their pharmacological mechanism. Systemic administration can trigger cytokine storms via the NF-κB and MAPK pathways. The TLR4 agonist LPS was limited in early clinical trials due to inducing sepsis-like reactions (Kanzler et al., 2007). Systemic administration of the TLR7/8 agonist resiquimod caused multi-organ inflammation in mouse models, accompanied by significant increases in IL-6, IL-12, and IFN-*γ* (Jurk et al., 2002). Clinical studies showed that the TLR9 agonist CpG 7909 combined with chemotherapy for non-small cell lung cancer was terminated early due to severe flu-like symptoms and hematological toxicity (Manegold et al., 2008). These findings have driven research strategies toward local administration and the development of biased agonists.
b) Population response variability: Individual responses to TLR agonists are influenced by multiple factors including genetic background, disease status, and microbiome. TLR4 gene polymorphisms (e.g., D299G) can blunt responses to LPS and are associated with increased infection risk (Arbour et al., 2000). The TLR7 gene is located on the X chromosome, leading to stronger antibody responses to TLR7 agonists in females than in males (Berghöfer et al., 2006). In cancer patients, the immunosuppressive state of the tumor microenvironment (e.g., enrichment of MDSCs and Tregs) significantly impairs the ability of TLR agonists to activate dendritic cells (Smith et al., 2018). These variations make it difficult to predict efficacy in clinical trials, highlighting the necessity for patient stratification and precision medicine.
c) Immune tolerance issues: Repeated TLR stimulation can induce homologous tolerance, manifested by downregulation of signaling molecules like IRAK and TRAF6, leading to reduced cytokine production (Dobrovolskaia and Vogel, 2002). In the tumor microenvironment, while TLR agonists can locally activate immunity, they are often accompanied by compensatory upregulation of immune checkpoints like PD-L1, limiting efficacy (Sistigu et al., 2014). These mechanisms explain the limited efficacy of monotherapy and support combination strategies with agents like immune checkpoint inhibitors.

## 7 Discussion

### 7.1 Current research gaps and unresolved issues

Despite significant progress in TLR adjuvant research, several gaps remain: (a) The characteristics of TLR responses in children and the elderly are unclear, as most existing data come from adult populations, and TLR expression levels and signaling efficiency differ in these age groups; (b) The impact of chronic underlying diseases (e.g., diabetes, autoimmune diseases) on the effectiveness of TLR adjuvants is unknown, as vaccine responses are often weaker in these populations; (c) The synergistic mechanisms between TLR agonists and novel vaccine platforms (e.g., viral vectors, DNA vaccines) are not fully elucidated, for instance, how co-delivery of TLR7 agonists with mRNA vaccines affects translation efficiency; (d) There is a lack of biomarkers to predict the safety of TLR adjuvants, currently still relying on clinical observation, unable to pre-identify high-risk populations.

### 7.2 Future directions

Integrating field development trends with existing gaps, future TLR adjuvant R&D should focus on three major directions: (a) Precision Adjuvant Design: Personalized adjuvants based on pathogen type and population characteristics, e.g., “TLR9 + TLR4” combination adjuvants for immunocompromised populations, TLR5 mucosal adjuvants for respiratory viruses; (b) Integration with Novel Delivery Systems: Developing “smart responsive carriers,” such as pH-sensitive LNPs that release TLR agonists only in the acidic environment of DC endosomes, further improving targeting; (c) Multi-Target Synergistic Strategies: Combining TLR agonists with agonists of other pattern recognition receptors (e.g., RIG-I, NOD2) to form an “immune enhancement network”; (d) AI-Assisted R&D: Utilizing AI to predict the structure–activity relationships of TLR agonists and personalized dosing regimens, shortening the R&D cycle.

## 8 Conclusion

TLR agonists, as vaccine adjuvants, can directionally enhance humoral and cellular immune responses through precise modulation of innate immune signaling pathways. Their clinical translation has achieved breakthrough progress (e.g., MPLA, CpG 1018). However, issues such as inflammatory toxicity and population response variability still limit their broad application. Strategies like targeted delivery, structural modification, and combination regulation can effectively mitigate risks. Current research needs to focus on the response characteristics of special populations like children and the elderly, as well as synergistic mechanisms with novel vaccine platforms. In the future, the integration of “precision design + smart delivery” will propel TLR adjuvants to leap from “universal-type” to “personalized,” providing new strategies for the development of novel antiviral vaccines.
